# Revealing redox isomerism in trichromium imides by anomalous diffraction†

**DOI:** 10.1039/d1sc04819h

**Published:** 2021-11-03

**Authors:** Amymarie K. Bartholomew, Rebecca A. Musgrave, Kevin J. Anderton, Cristin E. Juda, Yuyang Dong, Wei Bu, Su-Yin Wang, Yu-Sheng Chen, Theodore A. Betley

**Affiliations:** Department of Chemistry and Chemical Biology, Harvard University Cambridge MA 02139 USA betley@chemistry.harvard.edu; ChemMatCARS, The University of Chicago Argonne Illinois 60439 USA

## Abstract

In polynuclear biological active sites, multiple electrons are needed for turnover, and the distribution of these electrons among the metal sites is affected by the structure of the active site. However, the study of the interplay between structure and redox distribution is difficult not only in biological systems but also in synthetic polynuclear clusters since most redox changes produce only one thermodynamically stable product. Here, the unusual chemistry of a sterically hindered trichromium complex allowed us to probe the relationship between structural and redox isomerism. Two structurally isomeric trichromium imides were isolated: asymmetric terminal imide (^tbs^L)Cr_3_(NDipp) and symmetric, μ^3^-bridging imide (^tbs^L)Cr_3_(μ^3^–NBn) ((^tbs^L)^6−^ = (1,3,5-C_6_H_9_(NC_6_H_4_-*o*-NSi^*t*^BuMe_2_)_3_)^6−^). Along with the homovalent isocyanide adduct (^tbs^L)Cr_3_(CNBn) and the bisimide (^tbs^L)Cr_3_(μ^3^–NPh)(NPh), both imide isomers were examined by multiple-wavelength anomalous diffraction (MAD) to determine the redox load distribution by the free refinement of atomic scattering factors. Despite their compositional similarities, the bridging imide shows uniform oxidation of all three Cr sites while the terminal imide shows oxidation at only two Cr sites. Further oxidation from the bridging imide to the bisimide is only borne at the Cr site bound to the second, terminal imido fragment. Thus, depending on the structural motifs present in each [Cr_3_] complex, MAD revealed complete localization of oxidation, partial localization, and complete delocalization, all supported by the same hexadentate ligand scaffold.

## Abstract 2

Application of high-resolution Multiwavelength Anomalous Diffraction (MAD) allows the assignment of localized, partly delocalized, and fully delocalized oxidation in a series of trichromium imide isomers.

## Graphical abstract

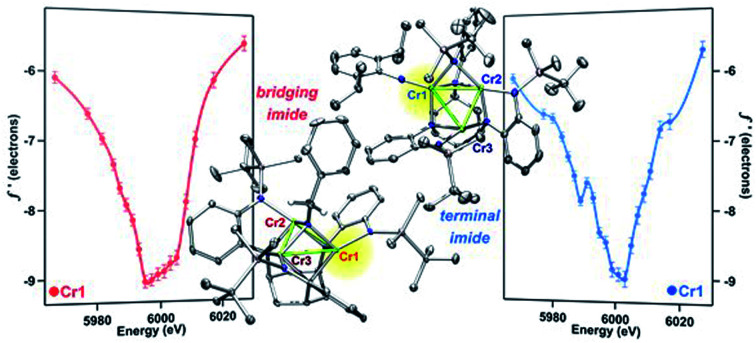

## Introduction

Transition metal clusters serve as biological enzyme active sites, homogeneous and heterogeneous catalysts, and landmark molecules in nanomaterial properties research.^1–4^ More specifically, synthetic transition metal clusters are important references for studies of complex bioinorganic systems, have been shown to mediate novel and challenging bond-activation reactions, and provide a means to develop fundamental understanding of metal–metal bonding, mixed-valency, and single-molecule magnetism.^5–9^ In such clusters, the relationship between geometric structure and electronic structure can be challenging to decipher due to the possible interplay of through-ligand superexchange, orbital-mediated direct exchange, and structural flexibility. Yet, determining how geometry affects delocalization is key to developing our understanding of diverse systems from ubiquitous biological iron sulfur clusters to the oxygen-evolving complex (OEC) of photosystem II and FeMoco of nitrogenase.^10–13^

Understanding how the geometry of a polynuclear cluster impacts delocalization is especially relevant as recent advances in the chemistry and crystallography of polynuclear biological active sites have proven that structural rearrangements are key to the function of FeMoco and the OEC of photosystem II, among others.^14–19^ Such rearrangements impact not only the docking and reactivity of substrates at the enzyme active sites, but also the pathways for electronic delocalization between the constituent metal atoms. Thus, furthering our knowledge of the relationship between geometry and delocalization in polynuclear transition metal clusters is not just a matter of fundamental scientific interest but also highly relevant to our understanding of biological active sites.

**Scheme 1:**
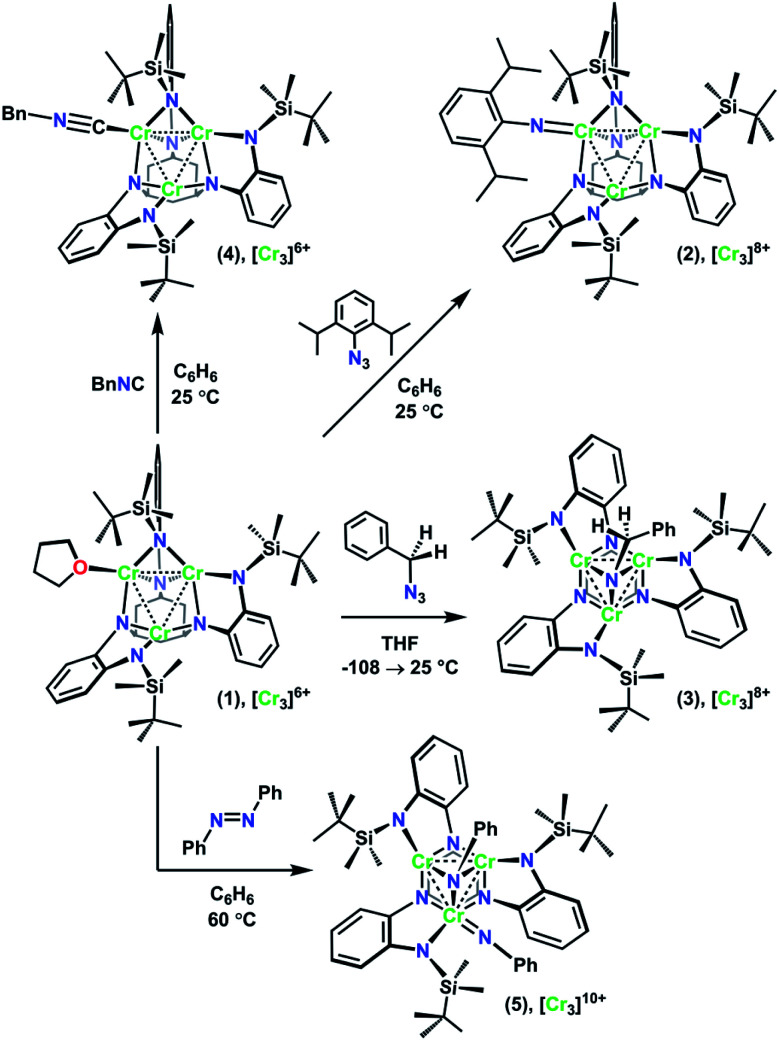
Synthesis of 2–5 from 1.

The recently reported chemistry of a trichromium complex on the hexadentate ligand ^tbs^LH_6_ (^tbs^LH_6_ = 1,3,5-C_6_H_9_(NHC_6_H_4_-*o*-NHSi^*t*^BuMe_2_)_3_) presented us with a unique opportunity to study the effects of geometric structure on the distribution of oxidation in two structurally isomeric, mixed-valent transition metal clusters.^20^ The all-chromous cluster (^tbs^L)Cr_3_(thf) (1) reacts with azides in several modes according to their steric bulk and the reaction medium. Changing the azide substrate size and reaction solvent provided us a path to isolate both the terminal imido complex (^tbs^L)Cr_3_(NDipp) (2) and the bridging imido complex (^tbs^L)Cr_3_(μ^3^–NBn) (3) (Dipp = (2,6-diisopropyl)phenyl, Bn = benzyl, Scheme 1). In addition to their structural isomerism, mixed-valency, and shared overall molecular oxidation state, these complexes feature bridging ligands capable of mediating superexchange and have metal–metal distances within the van der Waals radius of chromium, allowing for direct exchange between the metal centers.

In order to characterize the redox distribution in these two isomers, we turned to a technique that is particularly well-suited to the study of both structure and redox: Multiwavelength Anomalous Diffraction (MAD). MAD is a dynamic X-ray diffraction technique that utilizes a variable-wavelength, incident X-ray beam to examine the absorption profiles of each element within a material. The single crystal diffraction technique provides crystallographic, site-specific information analogous to that obtained from XANES spectroscopy on mononuclear species. Early examples of MAD were limited to a few data points per elemental K-edge of interest, providing limited utility for the resulting measurements due to the sensitivity of MAD to in-edge features.^21,22^ Recently, higher-resolution MAD studies have been conducted on synthetic manganese, iron, cobalt, and nickel-containing polynuclear clusters as well as on iron-containing biological cofactors such as FeMoco.^10,23–27^ A previous study by our research group revealed how cluster features (*e.g.*, metal primary coordination sphere, M–M bonding, mixed valency) in a triiron system impact MAD measurements, allowing us to correlate site specific redox information against bulk measurement techniques (*e.g.*, ^57^Fe Mössbauer, cyclic voltammetry).^28^

Alongside the two imide structure types 2 and 3, a homovalent structural analogue of 2, (^tbs^L)Cr_3_(CNBn) (4), and the further oxidized bisimide (^tbs^L)Cr_3_(μ^3^–NPh)(NPh) (5) were also investigated using MAD. The MAD results for this series of closely related trichromium complexes reveal distinct profiles of oxidation distribution for each of the three mixed-valent imide complexes, thus demonstrating the profound effects of geometric structure on redox load distribution even within complexes which are otherwise apparently similar.

## Results

### (I) Synthesis

As we have recently reported, the trichromium cluster (^tbs^L)Cr_3_(thf) (1) can readily undergo oxidative group transfer upon reaction with organic azides.^20^ The addition of 2,6-diisopropylphenyl azide (DippN_3_) to 1 affords the terminal imido (^tbs^L)Cr_3_(NDipp) (2); whereas the addition of benzyl azide to 1 yields the bridging imido product (^tbs^L)Cr_3_(μ^3^–NBn) (3) (Scheme 1). To provide a structurally similar, all-Cr^II^ reference compound to the terminal imido 2, the isocyanide adduct (^tbs^L)Cr_3_(CNBn) (4) was synthesized by benzylisocyanide addition to 1. While THF-adduct 1 is also an all-Cr^II^ complex, 1 is less structurally analogous to 2 than 4, as the Cr1−Cr2 distances in both the terminal imido 2 (2.7034(7) Å) and the isocyanide 4 (2.5541(6) Å) are elongated relative to the THF-adduct (2.439(1) Å).

Both 2 and 3 represent two-electron oxidations of the trichromium cluster (*e.g.*, [Cr_3_]^8+^, where [Cr_3_]^*n*+^ denotes the cumulative oxidation state of the trichromium core) compared to 1 ([Cr_3_]^6+^), but we have previously shown that the highly-reducing [Cr_3_] core of 1 is also capable of further substrate reduction to form four-electron oxidized (*e.g.*, [Cr_3_]^10+^) bis-imido products (^tbs^L)Cr_3_(μ^3^–NPh)(NMes) and (^tbs^L)Cr_3_(μ^3^–NBn)(NBn).^20^ The bis-imido complex (^tbs^L)Cr_3_(μ^3^–NPh)(NPh) (5) was selected for this study due to its straightforward synthesis and high crystallinity. Complex 5 was generated nearly quantitatively by addition of one equivalent of azobenzene (PhNNPh) to 1 in benzene solution at room temperature, followed by heating at 60 °C for 4 h (Scheme 1).

### (II) Solid-state molecular structures

In order to achieve reliable MAD data for small molecule crystals at a synchrotron source, several requirements must be met by the crystals. First, single crystals must be of a size and diffraction quality suitable for a publication using Mo Kα radiation at reasonable exposure times (*e.g.*, <30 s per frame). Second, the asymmetric unit of the crystal unit cell must contain all metal sites of interest. If any of the metal sites of interest are symmetry-equivalent in the unit cell, obtaining distinct atomic scattering factor values for those sites will not be possible. Lastly, structures containing multiple molecules of the cluster of interest in the asymmetric unit are to be avoided as the amount of data needed is proportional to the number of unique metal sites in the asymmetric unit.

Complexes 2–5 were first characterized by single-crystal, X-ray diffraction (SC-XRD) using a closed-source (Mo Kα), in-house diffractometer to ensure each sample was crystallographically well-suited for MAD analysis. In each instance, the asymmetric unit contained only one molecule of 2, 3, 4, or 5; and all three Cr sites in each molecule were located in the asymmetric unit. That the entire cluster was contained in the unit cell was especially important for bridging imide 3 as the most symmetric molecule of the series.

**Fig. 1:**
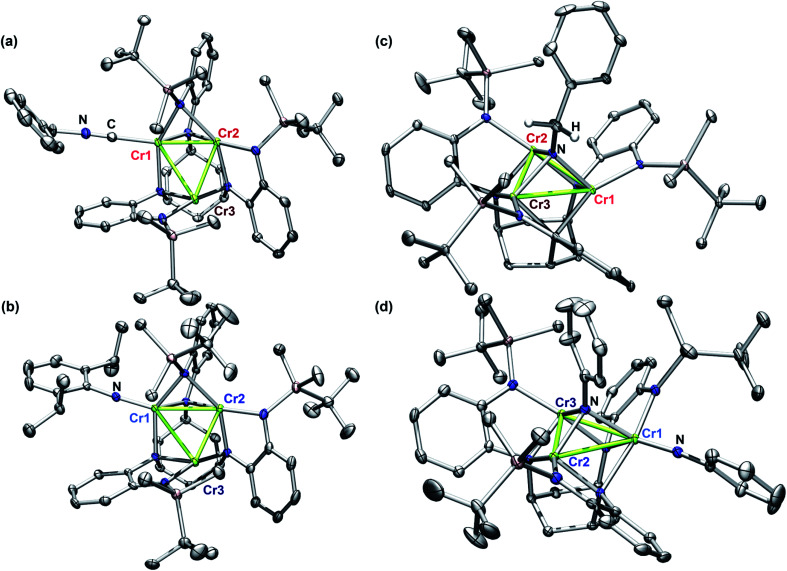
Solid state molecular structures of (a) (^tbs^L)Cr_3_(CNBn) (4); (b) (^tbs^L)Cr_3_(NDipp) (2); (c) (^tbs^L)Cr_3_(μ^3^–NBn) (3); and (d) (^tbs^L)Cr_3_(μ^3^–NPh)(NPh) (5). Structures are presented with ellipsoids at 50% and some H atoms have been omitted for clarity (C gray, N blue, Si Pink, H white, Cr green).

The solid-state structures of 2–5 collected at 30 keV (100 K) are shown in Fig. 1. The 30 keV structures obtained provide the positional references for subsequent refinement of data obtained near the Cr K-edge (5990 eV). Solid-state structures of 2 and 3 were previously reported, but further discussion of their bond metrics is warranted. Complexes 2 and 4 both feature the same asymmetric ligand binding mode as 1, with one four-coordinate Cr site (Cr1) bearing either THF, benzyl isocyanide, or NDipp; one four-coordinate ligand-bound site (Cr2); and one three-coordinate site (Cr3). As expected, the Cr1–NDipp distance of 1.681(2) Å in 2 is much shorter than the Cr1–CNBn distance of 2.031(2) Å in 4. The Cr1–NDipp distance in 2 is elongated relative to Cr–N_im_ of mononuclear Cr^IV^ imidos (1.64–1.67 Å), but shorter than those of known mononuclear Cr(iii) imidos (1.687(2) and 1.709(3) Å).^29,30^ Comparing 2 to its all-chromous structural analogue 4, the two-electron oxidation is subtly manifest in changes in the Cr–ligand (^tbs^L^6−^) distances, which are most easily summarized as the average Cr–N_ligand_ distance: Cr1–N_ligand_ and Cr2–N_ligand_ decrease by 0.03 and 0.07 Å, respectively, while Cr3–N_ligand_ increases by 0.07 Å.

Turning to a comparison of the structures bearing μ^3^-imido functionalities, 3 and 5 feature the same, pseudo-*C*_3_ symmetric binding mode of ^tbs^L^6−^. Each of the three chromium sites in both molecules is bound to two anilide units adjacent to the cyclohexyl backbone, one peripheral anilide, and the μ^3^-imide. The additional coordination of a second imide to Cr1 in 5 (Cr1–N_im_ 1.691(3) Å) is the only significant difference in coordination geometry between the two structures. While all three Cr sites of 3 feature the same coordination number and local geometry, Cr1 possesses a longer Cr–N_im_ bond length of 2.005(2) Å compared to both Cr2–N_im_ (1.954(2) Å) and Cr3–N_im_ (1.927(3) Å). From analysis of the bond-metrics alone it is unclear to what extent this difference reflects a difference in the redox states of the individual Cr sites. The bonds between the Cr sties and the bridging imide in 5 are similarly asymmetric: Cr1–N_im_, 2.030(3) Å; Cr2–N_im_, 1.946(2) Å; Cr3–N_im_, 1.923(2) Å, where the longest bond to the bridging imide is from the Cr site that also bears a terminal imide.

### (III) SQUID magnetometry

The spin states of complexes 1–5 were assessed by SQUID magnetometry. Each of the complexes displays a low-to-intermediate spin ground state indicating antiferromagnetic coupling between the individual Cr sites, which are anticipated to be intermediate-to-high spin.^31^ Despite the range of oxidation states featured, THF-adduct 1, terminal imide 2, isocyanide-adduct 4, and bis-imide 5 were found to all have triplet ground states. The bridging imide 3 is the lone exception, exhibiting a singlet ground state. For complexes 1, 3, and 4, the ground states observed are not well-isolated, with each showing population of excited spin states well below room temperature (Fig. S6, S9 and S7, respectively†). The *S* = 1 ground states of 2 and 5 are more well-isolated (Fig. S8 and S10†). Due to the remarkably low spin states of 1–5, it was not possible to derive much information about the electronic structure of individual Cr sites from the SQUID magnetometry of these complexes, further emphasizing the utility of MAD for assessing site-specific features, which can be difficult or impossible to discern from bulk measurements.

### (IV) Cyclic voltammetry and near-infrared absorption

The dramatically different primary coordination spheres of the Cr sites in terminal imido 2 and bis-imido 5 complicates the electrochemical assessment of redox delocalization by comproportionation constant. However, a comproportionation constant was determined for more symmetric imide 3.^32^ The cyclic voltammogram of 3 was collected in THF solution with tetrabutylammonium hexafluorophosphate as the supporting electrolyte (0.1 M) and subsequently referenced to [Cp_2_Fe]^0/+^. One reversible oxidation was observed at *E*_1/2_ = −0.893 V and one reversible reduction was observed at −1.26 V, followed by two additional irreversible reductions at lower potentials. This data was used to calculate 1.6 × 10^6^ as the upper limit of *K*_c_. This comproportionation constant is indicative of borderline class II/class III behavior according to the Robin Day classification.^33–35^ Cyclic voltammograms for 3 and 5 are presented in Fig. S5.†

The near-IR spectra of 2–5 were collected from 800 to 3300 nm to probe for the presence of intervalence charge transfer (IVCT) bands (Fig. S11†).^36^ While 2–4 absorb minimally in the near-IR, 5 displays a significant absorption peak at 8500 cm^−1^. A previously characterized mixed-valent bridging triiron imido on the same ligand platform and its homo-valent analogue were both observed to possess similar broad absorptions in the near-IR. In that case, the presence of an equivalent absorption in the homovalent, triiron imido complex ruled out the assignment of the transition as an IVCT.^28^ A homovalent structural analogue of 5 was not synthetically accessible, preventing the conclusive assignment of this feature as an IVCT.

### (V) Establishing redox references for MAD

As with other examples of MAD applied to synthetic molecules, the real component of the anomalous scattering factor (*f*′) is the focus of our data analysis, as the molecules of interest crystallize in centrosymmetric space groups.^37^ Given that this current study is the first application of MAD to chromium, we sought to use mononuclear Cr coordination complexes of well-defined oxidation states (Cr^II^ → Cr^IV^) to serve as references for the clusters examined. To provide reference benchmarks to the atomic scattering factors obtained by MAD, it is possible to derive the *f*′ factors from the corresponding X-ray absorption spectrum. The imaginary component of the anomalous scattering factor (*f*′′) is linearly related to the XAS spectrum of a complex. In turn, *f*′ and *f*′′ are related by a Kramers–Kronig relation:
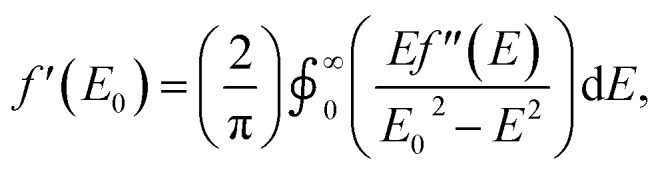
and a qualitative predicted *f*′ spectrum can therefore be obtained by applying a Kramers–Kronig transformation to the fluorescence edge scan of each reference complex.^38^ The application of this transformation to the edge scan is commonly used in applications of anomalous scattering in protein crystallography.^37^

**Fig. 2:**
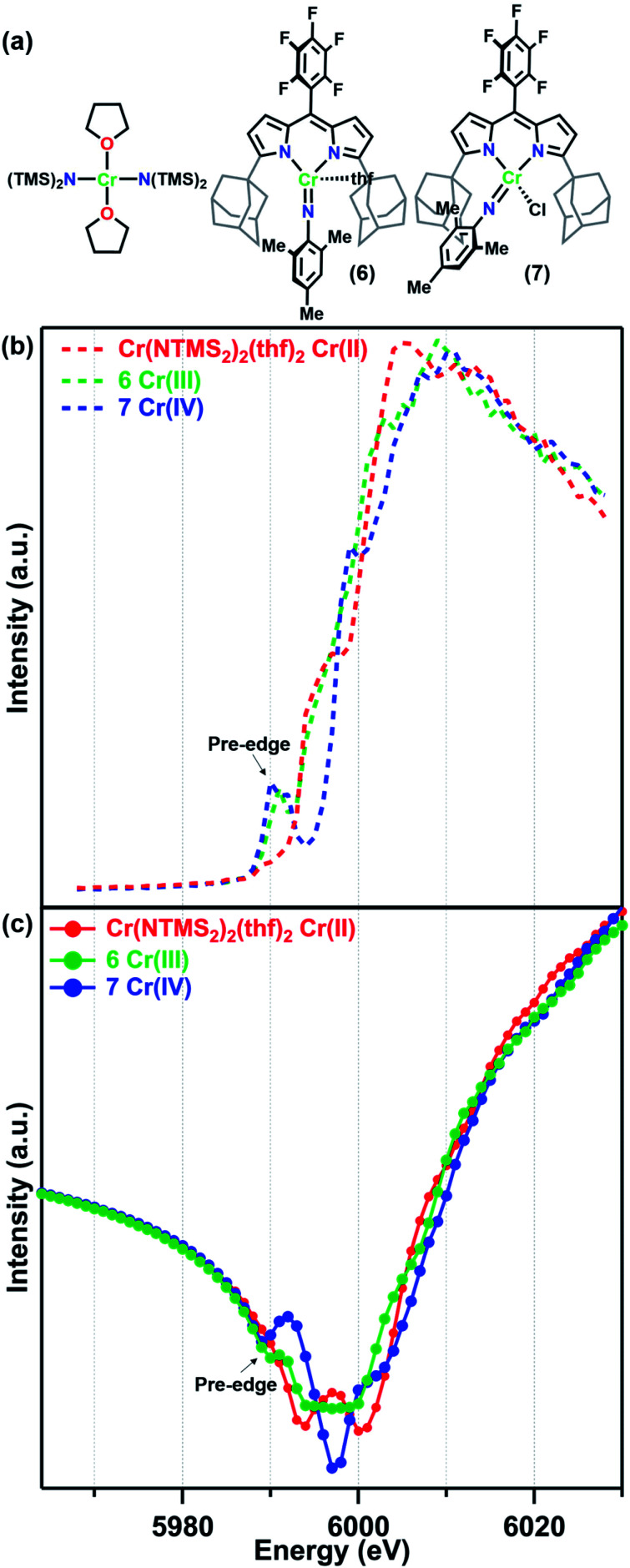
(a) Reference molecules Cr(NTMS_2_)_2_(thf)_2_, (^Ad^L_F5_)Cr(NMes)(thf) (6), and (^Ad^L_F5_)Cr(NMes)(Cl) (7) and their (b) fluorescence K-edge scans and (c) predicted *f*′ spectra.

For this study, the Cr K-edges of three reference molecules were assessed by X-ray fluorescence and subsequently transformed into *f*′ reference curves using a published MatLab toolbox for Kramers–Kronig transformations.^39^ For a Cr^II^ reference, the square planar amide Cr(N(TMS)_2_)_2_(thf)_2_ (TMS = trimethylsilyl) was used. Cr(N(TMS)_2_)_2_(thf)_2_ is known to possess a quintet ground state and Cr–N_amido_ bond lengths (2.04 Å)^31^ comparable to the Cr–N_ligand_ distances in 1–5. Mononuclear imido complexes previously synthesized by our group on a dipyrrin ligand platform (^Ad^L_F5_) were chosen as representative examples of the Cr^III^ and Cr^IV^ oxidation states (Fig. 2a).^30^ The Cr^III^ imido complex (^Ad^L_F5_)Cr(NMes)(thf) (6) features a four-coordinate Cr^III^ center in a distorted trigonal pyramidal geometry. The Cr^IV^ reference (^Ad^L_F5_)Cr(NMes)(Cl) (7) also features a four-coordinate Cr center, but in a tetrahedral geometry. Complexes 6 and 7 possess quartet and triplet ground states and Cr–N_im_ bond lengths of 1.671(9) and 1.640(5) Å, respectively.^30^

The Cr K-edge fluorescence scans and predicted *f*′ for Cr(N(TMS)_2_)_2_(thf)_2_, 6, and 7 are shown in Fig. 2. The intensities of the X-ray fluorescence scans cannot be normalized, but the resulting predicted *f*′ spectra have been scaled to have overlapping baseline curvature before the onset of the edge at 5990 eV (Fig. 2c). Nevertheless, the absolute intensities of the features should not be used for interpretation. Neither the Cr K-edge scan nor the predicted *f*′ spectrum for Cr(N(TMS)_2_)_2_(thf)_2_ displays a significant pre-edge feature. Prominent pre-edge features were observed in the Cr K-edges of both 6 and 7 as expected for imido complexes, where N 2p mixing from the imido can enhance the Cr 1s → 3d pre-edge feature.^40,41^ The predicted *f*′ spectra feature a corresponding pre-edge minimum at 5990 eV for 6 and 5989 eV for 7. The right-hand rising edge of 7 is shifted to slightly higher energies than those of the other two more reduced reference complexes.

Turning to the central portion of the predicted *f*′ spectra we see that the generated *f*′ spectrum of Cr^III^ imide 6 has a very broad minimum spanning roughly 10 eV. Meanwhile, Cr^IV^ imide 7 has a much sharper minimum at 5997 eV, corresponding to the on-edge feature in the K-edge scan, and the on-edge feature in the edge scan of Cr(N(TMS)_2_)_2_(thf)_2_ translates into a double-minimum in its *f*′ curve. As we have previously discussed for a related Fe system,^28^ the absolute minimum in *f*′ is strongly affected by in-edge features, which can arise from allowed 1s → 4p transitions or from “shake-down” transitions, depending on the local geometry of the metal site.^42,43^ Due to these in-edge features the absolute minimum of *f*′ should not be interpreted as directly related to oxidation state.

### (VI) Multiwavelength anomalous diffraction

We have previously reported a protocol for MAD which consistently resulted in well-resolved *f*′ curves for three unique sites of triiron complexes on the (^tbs^L) platform.^28^ Application of this protocol to the complexes in this study produced similarly well-defined *f*′ spectra to those in our previous report, despite the poorer diffraction expected at the lower-energy Cr K-edge (∼5990 eV) *vs.* that obtained at the Fe K-edge (∼7110 eV).

**Fig. 3:**
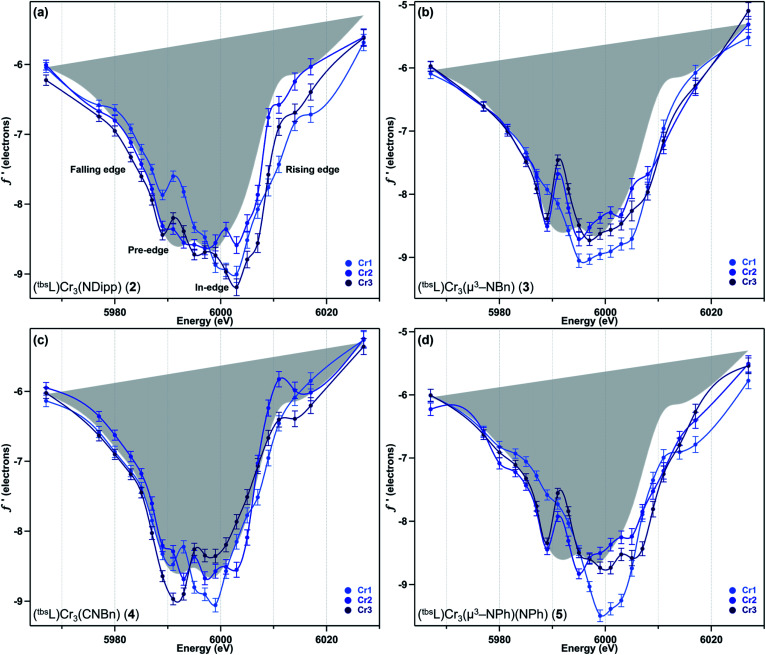
The experimental anomalous scattering factor *f*′ for each of the three Cr sites of (a) (^tbs^L)Cr_3_(NDipp) (2), (b) (^tbs^L)Cr_3_(μ^3^–NBn) (3), (c) (^tbs^L)Cr_3_(CNBn) (4), and (d) (^tbs^L)Cr_3_(μ^3^–NPh)(NPh) (5), with the average *f*′ for 4 shown in gray as a reference point for divalent chromium.

The collection strategy consisted of a full X-ray diffraction data collection at high energy (30 keV) as a structural reference, followed by a fluorescence scan of the Cr K-edge of the sample to determine suitable energies for MAD acquisition. Following the fluorescence scan, partial diffraction datasets at energies that span the K-edge of interest were collected. For samples 2, 4, and 5, 21 data points were collected from 5967–6027 eV. Due to time constraints, the collection for 3 was abbreviated to 18 data points redistributed throughout the same energy range. Each partial diffraction dataset included 500–1000 unique reflections. After integration of these reflections, the real (*f*′) and imaginary (*f*′′) scattering factors for each Cr were freely refined, while their position and occupancy were fixed at the values determined from the 30 keV diffraction dataset. For non-Cr atoms, tabulated scattering factors were used at each energy. The resulting *f*′ plots are shown in Fig. 3. The average of the three *f*′ curves of all-chromous 4 is included in gray on each of the *f*′ graphs to provide a general reference for the envelope of a typical Cr(ii) site for this ligand platform.

Terminal imide 2 was found to have a highly distinct *f*′ curve for each of its three unique Cr sites (Fig. 3a). Beyond the expected variation in the in-edge region due to the pronounced differences in local coordination geometry, the left-hand falling edges (5980–5990 eV) and right-hand rising edges (6005–6015 eV) of the three sites are also distinct. The *f*′ trace of imido-bearing Cr1 is shifted to the highest energy in the falling edge and shifted to higher energy than *f*′_Cr2_ in the rising edge. The *f*′ for three-coordinate Cr3 is shifted just slightly to higher energy than *f*′_Cr2_ in the falling edge and shifted 2 eV higher in energy than *f*′_Cr2_ in the rising edge. Both *f*′_Cr1_ and *f*′_Cr3_ display a pre-edge feature at 5989 eV.

In direct contrast to those in 2, the rising and falling edges of the three *f*′ curves of bridging imide 3 are closely overlaid (Fig. 3b). All three rising edges are shifted 3 eV higher in energy than the average *f*′ of 4, which is shown in gray as a reference for the envelope of *f*′ for a divalent chromium site within the (^tbs^L) ligand platform. While the overall shift to higher energies is comparable for all three sites of 3, *f*′_Cr1_ lacks the pronounced pre-edge feature at 5989 eV present in *f*′_Cr2_ and *f*′_Cr3_ (Fig. 3b).

Like its oxidized counterpart 2, asymmetric 4 displays three unique *f*′ curves (Fig. 3c). Though all three are nearly overlaid for the left-hand falling edge, they diverge beginning at 5989 eV. The differences between the three traces persist through the right-hand rising edge, with the three traces all crossing between 6004 and 6007 eV.

In bisimide 5, *f*′_Cr2_ and *f*′_Cr3_ remain similar in in-edge features and closely aligned through the falling and rising edge (Fig. 3d). The *f*′ curve of Cr1, the terminal imide-bearing site, is shifted to higher energies than *f*′_Cr2_ and *f*′_Cr3_ in the left-hand falling edge, but not in the rising edge. The shape of *f*′_Cr1_ consists of a single, narrow peak centred around 6000 eV without the pre-edge feature present in *f*′_Cr2_ and *f*′_Cr3_ at 5989 eV.

## Discussion

The (^tbs^L)Cr_3_ platform provided access to the unusual series of mixed-valent terminal imide 2, bridging imide 3 and mixed terminal and bridging bisimide 4, but the SQUID magnetometry and electrochemistry of these complexes were minimally informative with respect to any differences in redox distribution or delocalization. Comparison of the obtained *f*′ spectra of 2–4 provides insight into the differences in oxidation load distribution in the three formally mixed-valent imides investigated. Additionally, this study represents the first application of high-resolution MAD to chromium-containing clusters. Given that chromium has the lowest energy K-edge of the elements to which high resolution MAD has been applied, we note that *f*′ spectra were obtained with similarly low uncertainty values to those obtained for iron complexes following similar exposure times to synchrotron radiation (8–10 h per sample).^28^

The results from our study benchmarking the use of high-resolution MAD on a series of triiron complexes directly informed both this MAD study and our discussion and interpretation of its results, below. To summarize briefly, we determined that the local coordination geometry of each transition metal site in a polynuclear complex strongly impacts the appearance/features of the *f*′ curves, particularly in the pre-edge and in-edge regions. Rather than being indicative of the oxidation state, the shape of the curve was found to be primarily responsive to the ligand sphere and geometry. However, the left-hand falling edge and right-hand rising edge, *i.e.*, the overall energetic location of the *f*′ curve was found to shift to higher energy on oxidation.^28^ As in the previous study, the uncertainty in the incident X-ray energy is roughly 1 eV, so a shift ≤1 eV is within that uncertainty.

In light of studies by our group and others demonstrating the iminyl radical character of some NAr ligands^41,44–48^ and the impact this would have on characterization of the Cr oxidation states, it is necessary to discuss the nature of the NAr ligands bound to 2 and 5 before interpreting the MAD data. The most straightforward assessment of possible iminyl radical character for each NAr can be taken from the N–C_ipso_ distance, with iminyl radicals having much shorter distances (1.31–1.35 Å) than closed-shell imidos (1.37–1.42 Å).^44–46^ In 2, the NDipp N–C_ipso_ distance is 1.378(3) Å. In 5, the bridging NPh N–C_ipso_ distance is 1.420(4) Å and the terminal NPh N–C_ipso_ distance is 1.373(4) Å. While the N–C_ipso_ distances of the terminal imidos in 2 and 5 lie on the short end of closed-shell aryl imidos from the literature, it is worth noting that mononuclear Cr(iii) and Cr(v) imidos with very similar N–C_ipso_ distances (1.379(4) Å and 1.376(5) Å) lacked any nitrogen hyperfine coupling by EPR, leading to their unambiguous assignment as closed-shell imidos.^30^ By comparison to these literature structures we also assign the aryl imidos in 2 and 5 as closed-shell.

**Fig. 4:**
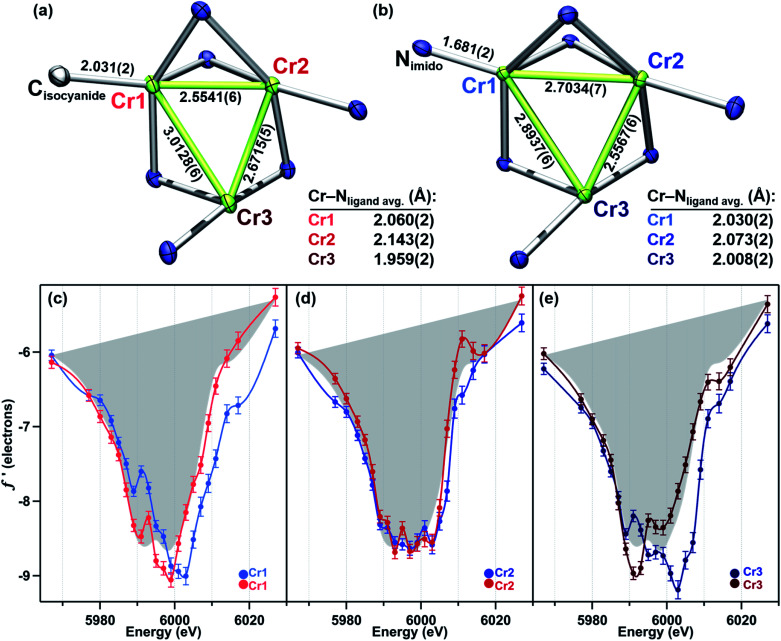
Crystal structure cores with bond metrics (Å) indicated of (a) (^tbs^L)Cr_3_(CNBn) (4), and (b) (^tbs^L)Cr_3_(NDipp) (2). (c–e) Direct comparison of the anomalous scattering factor *f*′ for (c) Cr1, (d) Cr2, and (e), Cr3 sites of 2 and 4 with the average *f*′ for 4 shown in gray as a reference point for divalent chromium.

Terminal imido cluster 2 and its homovalent analogue isocyanide complex 4 differ by the ligand terminally bound to site Cr1. The terminal imido ligation observed in 2 was the first example on the (^tbs^L) platform where cluster oxidation resulted in metal–ligand multiple bond formation as opposed to the imido functionality bridging the cluster in a μ^3^-fashion.^49^ We were interested in discerning whether this structural arrangement was reflective of redox localization to a single site within the cluster. However, a comparison of the *f*′ traces for 2 and 4 reveals the two-electron oxidation in 2 is not localized to Cr1 (Fig. 4c–e).

The 4 eV shift to higher energy in *f*′_Cr1_ going from 4 to 2 clearly indicates that oxidation has occurred at this site (Fig. 4c), as expected from substituting a terminal isocyanide for a terminal imido ligand. The extreme similarity in the shape of *f*′_Cr1_ for 4 to 2 is reflective of the conserved coordination geometry at this site, rendering the assignment of oxidation unambiguous. The overall shape of *f*′_Cr1_ in 4 and 2 also closely resembles the predicted *f*′ spectrum for Cr^IV^ imide reference 7. The anomalous scattering factors for Cr2 (*f*′_Cr2_) in both 2 and 4 overlay well (Fig. 4d), suggesting no oxidation state change at Cr2. A significant change in the shape and energy of *f*′_Cr3_ is observed between 4 and 2 (Fig. 4e). In particular, a pre-edge feature at the same energy as for Cr1 (5989 eV) emerges in *f*′_Cr3_ of 2, and the absolute minimum in *f*′_Cr3_ shifts to higher energy, as does the right-hand rising edge. The local geometry of Cr3 does not change significantly between the two structures, being T-shaped in both cases, but the Cr3–Cr1 and Cr3–Cr2 distances each contract by 0.12 Å while the Cr3–N_ligand_ bond length increases by 0.07 Å. The shifted energy of *f*′_Cr3_ and subtle pre-edge feature indicate some oxidation is borne at the Cr3 site. Furthermore, the Cr3–Cr1 contraction observed in 2 could result in the pre-edge feature observed, wherein the enhanced M–M bonding might increase the Cr pre-edge absorption in an analogous fashion to the terminal imido at Cr1. We conclude the two-electron oxidation in 2 is borne by Cr1, the site of the terminally bound imido, and, surprisingly, the distal Cr3 site.

**Fig. 5:**
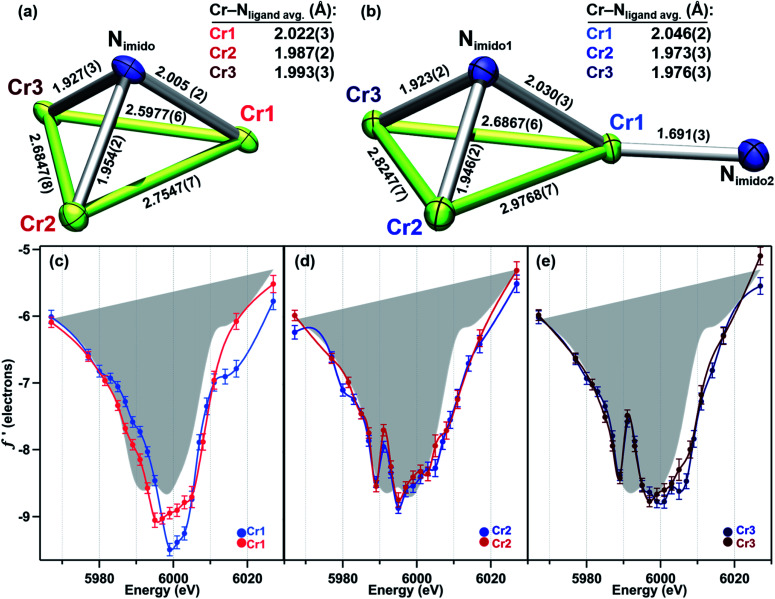
Crystal structure cores with bond metrics (Å) indicated of (a) (^tbs^L)Cr_3_(μ^3^–NBn) (3), and (b) (^tbs^L)Cr_3_(μ^3^–NPh)(NPh) (5). (c–e) Direct comparison of the anomalous scattering factor *f*′ for (c) Cr1, (d) Cr2, and (e), Cr3 sites of 3 and 5 with the average *f*′ for 4 shown in gray as a reference point for divalent chromium.

While the oxidation of the terminal imido cluster 2 appears to be distributed across two Cr sites, we anticipated that its symmetric structural isomer 3 might reveal oxidation equivalently distributed across the [Cr_3_]^8+^ core. Indeed, the MAD data confirms that all three Cr sites in 3 are comparably oxidized. The *f*′ curves of Cr2 and Cr3 (Fig. 5d and e) are extremely similar and closely related to the shape and pre-edge feature energy of the predicted *f*′ trace for the authentic Cr^III^ imide 6 (Fig. 2c). Despite the lack of pre-edge feature, Cr1 possesses an *f*′ curve which closely overlays with those of Cr2 and Cr3 throughout the left-hand falling and right-hand rising edges, which we have previously determined to be most reflective of oxidation state (Fig. 5c).^28^ The presence of a pre-edge feature in *f*′_Cr2_ and *f*′_Cr3_ but its absence in *f*′_Cr1_ can be explained by the Cr–N_imido_ bond metrics of 3. Cr1 possesses a Cr–N_imido_ bond that is >0.05 Å longer than those of Cr2 and Cr3 (2.005(2) Å *vs.* 1.954(2) Å and 1.927(3) Å, respectively). We note the all-ferrous chloride-capped and imide-capped triiron clusters with a similarly isosceles [Fe_3_(μ^3^–cap)] binding geometry similarly displayed two identical *f*′ curves and one with a comparable energetic envelope but distinct shape.^28^ The comproportionation constant (*K*_c_) of 1.6 × 10^6^ observed for 3 is consistent with the MAD findings in that it indicates significant delocalization (class II/III borderline).

As a combination of the bridging and terminal imido motifs seen in isomers 2 and 3, we were interested in how further oxidation was borne within the 4-electron oxidized bisimido cluster 5. The scattering factors *f*′_Cr2_ and *f*′_Cr3_ in 5 are unaffected by the addition of a second imide and produce identical traces both to each other as well as to their counterparts in 3 (Fig. 5d and e). Akin to 2, the trace for terminal imido-bound Cr1 (*f*′_Cr1_) shows signs of oxidation, particularly in the shift to higher energy of the left-hand side of the scattering factor curve (Fig. 5c). The narrowing of the *f*′ trace is also consistent with the predicted *f*′ trace for Cr^IV^ reference 7. Surprisingly, *f*′_Cr1_ lacks a pre-edge absorption in stark contrast to the features found in the predicted *f*′ spectra of mononuclear imido references 6 and 7, as well as *f*′_Cr1_ for 2 which bears the terminal imide. The Cr1–N_imido_ bond length of 1.691(3) Å is consistent with metal–ligand multiple bonding, which we anticipated would manifest a pre-edge absorption. We do note that Cr1–N_imido_ is elongated compared to Cr–N_imido_ bonds in mononuclear Cr^IV^ imido complexes. We cannot rule out the additional possibility that the pre-edge absorption is sharp and that an even higher resolution MAD study than that presented here would be necessary to observe the pre-edge feature in *f*′_Cr1_.

Unlike in 2, the primary manifestation of oxidation at Cr1 in 5 is in the left-hand falling edge of the *f*′ trace, rather than in both the left and right-hand edges of the trace. There are several plausible explanations for this observation – some of the oxidation may be delocalized onto the NAr fragment, which does possess an N_imido_–C_ipso_ bond on the short end for characterized closed-shell aryl imidos, some of the oxidation may be borne on the redox-active *ortho*-phenylenediamine (OPDA) ligand arm chelated to Cr1, and, lastly, the oxidation may be fully borne by Cr1, but for the particular metal and local coordination environment, only result in the observed shift in the left-hand side of the *f*′ trace. The bond metrics of the OPDA bound to Cr1 are not significantly perturbed compared to the other two OPDA arms found in 5, thus it is unlikely that ligand oxidation has occurred. Based on our discussion of the NPh bond metrics above, it is also unlikely that the NPh fragment is only partly reduced, leading us to tentatively conclude that this particular Cr-centered oxidation only results in a shift of the left-hand side of the *f*′ trace to higher energy.

We note that while a nitrogen K-edge XANES study could potentially clarify both the absence of a pre-edge feature at this site and the possible partial reduction of the imido, the presence of multiple imido fragments bound in distinct geometries would likely complicate the interpretation of the results, similar to the difficulties encountered in assessing the polynuclear complexes by other bulk measurements. Regardless of the extent of oxidation at Cr1, it is clear that in 5 no oxidation is borne at Cr2 or Cr3, making this the only species in this study for which further oxidation is localized at a single Cr site.

## Conclusions

Using multiwavelength anomalous diffraction, we assessed the distribution of oxidation in three imido-bound isomers of a trichromium complex. In terminal imide 2, redox is localized to two Cr sites with the third Cr site unchanged *vs.* homovalent analogue 4. In bridging imide 3, minor asymmetry in the Cr–N_imido_ bond lengths is reflected in the shape of *f*′ rather than in the location of the *f*′ envelope, leading us to conclude that all three sites are oxidized. Four-electron oxidized bis-imide 5 undergoes the most localized oxidation – only the terminal imido-bound site is oxidized compared to 3.

The foregoing study adds to the growing literature demonstrating the utility of MAD for oxidation state assessment of polynuclear transition metal clusters^10,23–28^ and presents the utility of absorption edge-generated *f*′ curves of mononuclear references. Furthermore, the complete overlay of the *f*′ traces for Cr2 and Cr3 of 3 and 5 despite a difference of two-electrons in the overall [Cr_3_] oxidation state of 5*vs.*3 ([Cr_3_]^10+^*vs.* [Cr_3_]^8+^) provides excellent evidence that this technique and data collection strategy is truly sensitive to the local metal oxidation state and unaffected by the aggregate oxidation state of the polynuclear core. Taking these results together with those from our previous work on Fe_3_ clusters, we conclude that a shift of ≥2 eV in the falling or rising edge of *f*′ represents a change in oxidation state at that site, so long as this shift is benchmarked against a metal site with similar geometry.

The (^tbs^L)Cr_3_ system shows that a single polynuclear complex can achieve localized or delocalized mixed-valent states based on the geometry of substrate binding and reactivity, rather than being structurally predisposed towards one regime or the other. The finding that terminal imide 2 is oxidized at both the imido-bearing site and at a second Cr site highlights that within a polynuclear cluster oxidation can be shared even when activated substrates are bound terminally to one site. Yet, the same system achieves complete localization of the two-electron oxidation from 3 to 5 at only the terminal imido-bound site. The flexible nature of the redox distributions found for the (^tbs^L)Cr_3_ imides points to the ability of a single polynuclear cluster to localize, partly delocalize, or fully delocalize redox equivalents depending on structural arrangements. A given polynuclear active site in biology may similarly be able to achieve localized or delocalized redox states dependent upon geometry, and analysis of the emerging importance of structural rearrangements in polynuclear active sites should proceed with this in mind.

## Data availability

Crystallographic data for 4 and 5 has been deposited at the CCDC (1949233, 1949234) and can be obtained from DOI: 10.1039/d1sc04819h. Crystallographic and refinement details, spectroscopic characterization, magnetic susceptibility, and CheckCIF results are provided in the ESI.†

## Author contributions

AKB and TAB conceived of this study. MAD data collection was performed by AKB, RAM, KJA, and CEJ with oversight and assistance from WB, S-YW, and Y-SC. All other synthesis and characterization was performed by AKB, aside from the synthesis of mononuclear reference compounds, which was carried out by YD. MAD data workup was performed by AKB and RAM. Data analysis was performed by AKB and TAB, and the manuscript was written by AKB and edited by TAB and RAM.

## Conflicts of interest

There are no conflicts to declare.

## Supplementary Material

SC-012-D1SC04819H-s001

SC-012-D1SC04819H-s002
