# Supplementary material for: Revealing redox isomerism in trichromium imides by anomalous diffraction
Source: Chem Sci. 2021 Nov 3;12(47):15739–49. doi: 10.1039/d1sc04819h (PMC8654065; doi:10.1039/d1sc04819h)
Supplement: SC-012-D1SC04819H-s001 [file SC-012-D1SC04819H-s001.pdf]

Electronic Supplementary Material (ESI) for Chemical Science.
This journal is © The Royal Society of Chemistry 2021

Supplementary Information for:

Revealing Redox Isomerism in Trichromium Imides by Anomalous Diffraction

Amymarie K. Bartholomew†, Rebecca A. Musgrave†, Kevin J. Anderton†, Cristin E. Juda†, Yuyang Dong†, Wei Bu§, Su-Yin Wang§, Yu-Sheng Chen§, and Theodore A. Betley*

†Department of Chemistry and Chemical Biology, Harvard University, 12 Oxford Street, Cambridge, Massachusetts 02138, United States

§ChemMatCARS, The University of Chicago, Argonne, Illinois 60439, United States

E-mail: betley@chemistry.harvard.edu

Table of Contents

Experimental methods S2

Table S1. Crystallographic data for (^{tbs}L)Cr_3(CNBn) (**4**) and (^{tbs}L)Cr_3(μ^3–NPh)(NPh) (**5**). S8

Table S2. Statistics of the anomalous diffraction data refinement for (^{tbs}L)Cr_3(CNBn) (**4**). S9

Table S3. Statistics of the anomalous diffraction data refinement for (^{tbs}L)Cr_3(NDipp) (**2**). S10

Table S4. Statistics of the anomalous diffraction data refinement for (^{tbs}L)Cr_3(μ^3–NBn) (**3**). S11

Table S5. Statistics of the anomalous diffraction data refinement for (^{tbs}L)Cr_3(μ^3–NPh)(NPh) (**5**). S12

Table S6. Comparison of relevant structural metrics. All bond metrics are reported in Å. S13

Figure S1. Fluorescence scans for **4** (red) and **2** (blue). S14

Figure S2. Fluorescence scans for **3** (red) and **5** (blue). S15

Figure S3. Anomalous scattering f'' plots for **4** (top) and **2** (bottom). S16

Figure S4. Anomalous scattering f'' plots for **3** (top) and **5** (bottom). S17

Figure S5. Cyclic voltammograms of **3** and **5** in 0.1 M [NBu_4][PF_6] in THF at a 0.05 V/s scan rate, referenced to ferrocene$^{0/+}$. S18

Figure S6. Variable-temperature magnetic susceptibility of **1**. S19

Figure S7. Variable-temperature magnetic susceptibility of **4**. S20

Figure S8. Variable-temperature magnetic susceptibility of **2**. S21

Figure S9. Variable-temperature magnetic susceptibility of **3**. S22

Figure S10. Variable-temperature magnetic susceptibility of **5**. S23

Figure S11. UV-Vis/Near-IR spectra of **2** (green), **3** (blue), **4** (red), and **5** (purple) at 298 K in THF. S24

Figure S12. Overalay of edge-scan derived predicted *f'*spectra for mononuclear references with the average *f'* of (^{tbs}L)Cr_3(CNBn) (gray). S25

Figure S13. Bond metrics for the imido fragment of **2**. S26

Figure S14. Bond metrics for the imido fragments of **5**. S26

References S27

Experimental methods

General considerations

All manipulations involving metal complexes were carried out using standard Schlenk or glovebox techniques under a dinitrogen atmosphere, unless otherwise noted. All glassware was oven-dried for a minimum of 10 h and cooled in an evacuated chamber prior to use in the drybox. Diethyl ether, hexanes, benzene, toluene, and tetrahydrofuran (THF) were dried and deoxygenated on a Glass Contour System (SG Water USA, Nashua, NH) and stored over 4 Å molecular sieves (Strem) prior to use. C_6D_6 was purchased from Cambridge Isotope laboratories and stored over 4 Å molecular sieves prior to use. Non-halogenated solvents were frequently tested, by a solution of sodium benzophenone ketyl in THF, for effective water and dioxygen removal. Celite 545 (J. T. Baker) was dried in a Schlenk flask for at least 20 h under dynamic vacuum while heating to 200 – 220 °C prior to glovebox use. (^{tbs}L)Cr_3(thf), (^{tbs}L)Cr_3(NDipp), and (^{tbs}L)Cr_3(μ^3–NBn) were synthesized according to procedures previously reported by our laboratory (see Chapter 3). PhN_3 was prepared by diazotization of the corresponding aniline and subsequent treatment with NaN_3. All organic azides were dissolved in hexanes and stored over 4 Å molecular sieves prior to use, at which point the solution was decanted and the hexanes was evaporated. All other reagents were purchased from commercial vendors and used without further purification.

Electrochemical measurements

Cyclic voltammetry was performed with a CHI660d potentiostat using a three-electrode cell with a glassy carbon working electrode and a platinum wire as the counter electrode. All measurements were conducted using a Ag/AgCl pseudoreference consisting of a silver wire immersed in 0.1 M [^{n}Bu_4N][PF_6] electrolyte and separated from the working compartment by a porous CoralPor (BASi) frit. All potentials are referenced to the ferrocene/ferrocenium (Fc/Fc+)

couple, which was measured at the end of every experiment by adding a small amount of ferrocene to the electrolyte solution.

Magnetic susceptibility measurements

Magnetic data for **1-5** were collected using a Quantum Design MPMS-XL Evercool SQUID Magnetometer. A general procedure for sample preparation is as follows: microcrystalline material was dried thoroughly in vacuo overnight and then crushed to a fine powder in an agate mortar and pestle. This crushed powder was then immobilized within a gelatin capsule size #4 by adding melted eicosane at 50 – 60 °C. The gelatin capsule was then inserted into a plastic straw. Samples were prepared under a dinitrogen atmosphere. Magnetization data at 100 K from 0 to 7 T was used as a ferromagnetic-free purity test. Direct current (dc) variable temperature magnetic susceptibility measurements were collected in the temperature range 5–300 K under applied fields of 0.5 and 1 T. Magnetization data were acquired at 2 – 10 K at fields 1, 4, and 7 T. Magnetic susceptibility data was corrected for diamagnetism of the sample, estimated using Pascal's constants, in addition to contributions from the sample holder and eicosane. The magnetic susceptibility data was collected multiple times until at least two different batches reproduced the data. Reduced magnetization data was modeled in PHI[1] according to the spin Hamiltonian: $\hat{H}$ = DSz2 + E(Sx2 – Sy2) + gμBS·B.

Other physical measurements

^{1}H NMR spectra were recorded on Agilent DD2 600 MHz or Varian Unity/Inova 500 MHz spectrometers. Chemical shifts for ^{1}H are reported relative to $SiMe_4$ using the chemical shift of residual solvent peaks as reference. Elemental analyses (%CHN) were obtained using a PerkinElmer 2400 Series II CHNS/O Analyzer.

Synthesis

(^{tbs}L)Cr$_3$(CNBn) (4). A 5% weight solution of benzyl isocyanide (CNBn) (241.9 mg, 0.103 mmol) in benzene was further diluted by the addition of 1 mL of benzene. This dilute solution was then added dropwise at r.t. to a stirred solution of *(^{tbs}L)Cr$_3$(thf) (1)* (100 mg, 0.103mmol) in benzene (3 mL). After stirring for 1 h at room temperature the magnetic stir bar was removed and the solution was frozen and removed in vacuo. This yielded *(^{tbs}L)Cr$_3$(CNBn) (4)* (94.5 mg, 0.093 mmol, 90% yield) as a brown powder. Crystals suitable for X-ray diffraction were grown from a concentrated diethyl ether solution at -35 °C. ^{1}H NMR (500 MHz, C_6D_6, δ, ppm): 59.94(b), 56.40(b), 42.90(b), 21.90(b), 16.93, 13.68, 3.46, 2.27, 1.95, 0.55, -0.86, -5.72. Anal. Calc. for $C_{50}H_{73}Cr_3N_7Si_3$: C, 59.32; H, 7.27; N, 9.68. Found: C, 59.36; H, 7.24; N, 9.61.

(^{tbs}L)Cr$_3$(μ^3–NPh)(NPh) (5). A concentrated solution of *(^{tbs}L)Cr$_3$(thf) (1)* (106.9 mg, 0.111 mmol) in 1 mL of benzene was added to solid azobenzene (PhNNPh, 20.1 mg, 0.111 mmol). An immediate color change from green-brown to red was observed. The solution was then transferred to a J. Young NMR tube, which was sealed under nitrogen, removed from the glovebox, and heated to 60 °C for four hours. After cooling to room temperature, the reaction vessel was returned to the glovebox and the solution was transferred to a scintillation vial and dried in vacuo. This yielded *(^{tbs}L)Cr$_3$(μ^3–NPh)(NPh) (5)* as a red-brown powder (102.5 mg, 0.095 mmol, 85.7% yield). Crystals suitable for X-ray diffraction were grown from a concentrated diethyl ether solution at -35 °C. ^{1}H NMR (500 MHz, C_6D_6, δ, ppm): 86.86(b), 60.83(b), 36.72, 20.41, 16.57, 13.95, 12.59, 12.19, 8.60, 6.75, 5.94(b), 3.04, 1.80, 1.09, 0.78, -1.35, -6.81, -9.15, -27.05. Anal. Calc. for $C_{54}H_{76}Cr_3N_8Si_3$: C, 60.19; H, 7.11; N, 10.40. Found: C, 59.9; H, 6.92; N, 10.20.

X-ray structure determinations.

Single crystals suitable for X-ray structure analysis were coated with deoxygenated Paratone N-oil and mounted in MiTeGen Kapton loops (polyimide). Data for compounds **2** - **5** was collected at 100 K using synchrotron radiation at the Argonne National Laboratory Advance Photon Source, ChemMatCARS. A full dataset suitable for structure determination was collected at 100 K using 30 keV radiation. The single crystals of **2** - **5** did not show decay during data collection. Data was collected using a Bruker three-circle platform goniometer equipped with a Bruker APEX II CCD and an Oxford Cryosystems cooling apparatus. The collection method involved 0.5° scans in ϕ at –5° in 2θ. Data integration down to at least 0.84 Å resolution was carried out using SAINT V8.34 C (Bruker diffractometer, 2014) with reflection spot size optimization. Absorption corrections were applied using SADABS.[2] The structure was solved by the Intrinsic Phasing methods and refined by least-squares methods again F^2 using SHELXL[3] with the OLEX 2[4] interface. Space group assignments were determined by examination of systematic absences, E-statistics, and successful refinement of the structures. The program PLATON[5] was employed to confirm the absence of higher symmetry. Non-hydrogen atoms were refined with anisotropic displacement parameters, and hydrogen atoms were added in idealized positions and refined using a riding model. Crystallographic data for **4** and **5** are given in Table S1.

Multiple-wavelength anomalous diffraction.

Energy referencing.

The energy of the beamline at Argonne National Laboratory Advance Photon Source, ChemMatCARS, was referenced by performing a fluorescence scan of the Fe K-edge of a single crystal of sublimed ferrocene in steps of 1 eV using a Vortex-EX 3070 silicon drift diode detector.

The ferrocene K-edge obtained this way was then compared to an XAS K-edge of sublimed ferrocene obtained at the Stanford Synchrotron Radiation Lightsource and the energy of the beamline was adjusted so that the energies of the pre-edge and rising edge as determined by both techniques were well aligned. All Fe XAS K-edge data collected at the Stanford Synchrotron Radiation Lightsource was referenced to iron foil.

Data collection strategy.

The following general procedure was followed for all samples: first, the crystal was mounted when the beam energy was at 30 eV. At this energy the crystal quality was judged by checking for the presence of twin domains. The crystal of each molecule which was both most single and gave the highest resolution at lowest exposure times was then selected to be used for the collection. A full structure diffraction dataset was then collected at this energy (30 keV). Second, the beam energy was lowered to the Cr K-edge and a fluorescence scan (Figure 2c top and S4 top) was collected from 5939 to 6939 eV in steps of 1 eV by using a Vortex-EX 3070 silicon drift diode detector. This data was used to determine the energies at which to collect the partial diffraction data. Third, partial diffraction data was collected as described below at increasing energy.

For **2**, **4**, and **5**, data was collected at 21 energies between 5967 and 6027 eV. For **3**, data was collected at 18 energies between 5967 and 6027 eV. For **2**-**5**, a total of 480 frames were collected at each energy using 4 pairs of φ scans with 2θ angles of -10°, -30°, -60°, and -90° for each pair and ω angles of 180° and 220° for the two scans within each pair. The step size in ϕ was 0.5° for all scans.

Anomalous diffraction data refinement.

The 30 keV data was modeled to provide structural data on the compound of interest. This model was used as the reference for each partial diffraction dataset as is described below. For the data integration of the highest energy anomalous diffraction data set, the unit cell parameters of the reference (.p4p file) were imported into APEX3 to provide a starting point. For subsequent energy points, a .p4p file of the preceding energy point was imported instead. A starting box size for the refinement was estimated based on the size of a single diffraction spot and refined during integration. A static mask was generated using AMask and employed to correct for the effects of the beamstop during integration. Integration was performed iteratively, reimporting the .p4p file after each run, until the unit cell parameters stabilized. For a single energy, frames with unique values of 2θ were integrated independently using the unit cell from an integration at the same energy and similar 2θ values wherever possible. After integration, the data was scaled in APEX3 using the default settings. The .hkl and .ins files were generated with XPREP, setting the space group to match the one from the 30 keV reference. This procedure resulted in about 600 unique reflections at each energy step. Subsequently, the contents of the .ins file of the reference structure were copied into the .ins file generated for the anomalous diffraction datasets with the exception of the CELL and ZERR lines. The SFAC line of each .ins file was adjusted to reflect the number of unique metal sites. The atom type indices were then adjusted to account for this change to the SFAC line. At this point every one of the anomalous diffraction data sets had an associated .hkl and .ins file suitable for refinement in JANA2006.[6] These two files were imported into JANA2006. Using JANA2006 the scattering factors and were freely refined for each chromium site while keeping all structural parameters constant (using the FIXEDALL command). The anomalous scattering factors, and the corresponding errors (given as 2σ), obtained are tabulated in Tables S3-6 plotted in Figures S3-6.

Table S1. Crystallographic data for (^{tbs}L)Cr_3(CNBn) (**4**) and (^{tbs}L)Cr_3(μ^3–NPh)(NPh) (**5**).

	(^{tbs}L)Cr_3(CNBn) (**4**)	(^{tbs}L)Cr_3(μ^3–NPh)(NPh) (**5**)
**Chemical formula**	$C_{56}H_{79}Cr_3N_7Si_3$	$C_{58}H_{86}Cr_3N_8OSi_3$
**Formula weight**	1090.53	1151.61
**Space group**	$P2_1/c$	$P\bar{1}$
a **(Å)**	13.3541(5)	12.4743(5)
b **(Å)**	29.4601(11)	14.4695(7)
c **(Å)**	14.0800(5)	17.6459(8)
α **(deg)**	90	104.1910(10)
β **(deg)**	93.2710(10)	90.5520(10)
γ **(deg)**	90	105.9240(10)
V **(Å^3)**	5530.2(4)	2959.2(2)
Z	4	2
**μ (mm^{-1})**	0.362	0.154
**T (K)**	100	100
**GOF (S) [all data]**	1.031	1.024
***R*1^a (*wR*2^b) [*I*>2σ(*I*)]**	0.0399 (0.1025)	0.0492 (0.1084)
***R*1^a (*wR*2^b) [all data]**	0.0550 (0.1110)	0.0765 (0.1240)
**Reflections**	12565	13259
**Radiation type**	Synchrotron	Synchrotron

$^a R1 = \Sigma[w(F_o - F_c)]/\Sigma[wF_o]$; $^b wR2 = [\Sigma[w(F_o^2 - F_c^2)^2]/\Sigma[w(F_o^2)^2]]^{1/2}$, $w = 1/[\sigma^2(F_o^2) + (aP)^2 + bP]$, where $P = [\max(F_o^2,0) + 2(F_c^2)]/3$

Table S2. Statistics of the anomalous diffraction data refinement for (^{tbs}L)Cr_3(CNBn) (**4**).

	**Cr1**				**Cr2**				**Cr3**				**Reflections**		R_{obs}	wR_{all}
Energy (eV)	f'	esd	f''	esd	f'	esd	f''	esd	f'	esd	f''	esd	**Obs.**	**Total**		
5967	-6.14	0.16	1.62	0.36	-5.95	0.15	0.29	0.55	-6.02	0.16	1.01	0.50	487	505	5.48	7.29
5977	-6.58	0.16	1.73	0.31	-6.36	0.14	0.07	0.43	-6.63	0.15	0.80	0.42	489	502	5.38	7.19
5980	-6.86	0.16	1.74	0.30	-6.63	0.14	-0.27	0.42	-6.90	0.15	0.59	0.42	491	507	5.15	7.61
5983	-7.14	0.16	1.58	0.31	-6.93	0.15	0.30	0.40	-7.19	0.15	1.37	0.37	488	508	5.46	7.49
5985	-7.38	0.16	1.55	0.29	-7.18	0.14	0.12	0.38	-7.45	0.15	1.23	0.39	495	509	5.68	7.75
5987	-7.85	0.18	0.41	0.47	-7.60	0.19	1.54	0.39	-8.03	0.18	0.30	0.63	379	384	5.90	8.24
5989	-8.33	0.16	0.86	0.36	-8.21	0.17	1.95	0.29	-8.64	0.16	0.67	0.43	504	514	5.86	7.97
5991	-8.47	0.17	2.09	0.31	-8.29	0.17	0.99	0.35	-8.97	0.17	2.19	0.34	502	510	6.16	8.45
5993	-8.22	0.17	2.00	0.37	-8.69	0.17	2.00	0.36	-8.90	0.17	2.96	0.35	498	511	5.89	7.43
5995	-8.80	0.17	2.06	0.35	-8.36	0.18	2.34	0.33	-8.26	0.17	3.11	0.32	504	514	6.17	7.99
5997	-8.90	0.18	2.60	0.38	-8.67	0.19	2.74	0.36	-8.35	0.18	3.23	0.35	504	516	5.98	7.67
5999	-9.06	0.19	3.34	0.38	-8.57	0.20	2.91	0.38	-8.35	0.20	3.61	0.38	500	515	6.36	7.90
6001	-8.57	0.21	4.05	0.35	-8.51	0.21	3.31	0.35	-8.19	0.20	3.98	0.34	502	518	6.44	8.31
6003	-8.15	0.21	4.33	0.35	-8.55	0.21	3.83	0.35	-7.87	0.21	4.23	0.34	503	520	6.41	8.30
6005	-7.77	0.21	4.38	0.36	-8.09	0.22	4.84	0.35	-7.51	0.21	4.45	0.35	504	522	6.44	7.98
6007	-7.51	0.22	4.66	0.39	-7.03	0.23	5.14	0.38	-7.07	0.22	4.67	0.36	500	523	6.36	7.76
6009	-6.95	0.23	4.89	0.38	-6.24	0.23	4.86	0.40	-6.67	0.22	4.66	0.37	507	524	6.43	7.97
6011	-6.46	0.22	4.77	0.37	-5.82	0.22	4.31	0.41	-6.40	0.21	4.49	0.36	503	524	6.33	7.71
6014	-6.09	0.23	4.49	0.42	-5.98	0.23	3.95	0.47	-6.39	0.23	4.47	0.40	497	525	6.63	7.91
6017	-5.85	0.23	4.47	0.41	-6.02	0.23	3.79	0.46	-6.20	0.23	4.47	0.40	501	526	6.84	8.40
6027	-5.26	0.24	4.10	0.45	-5.25	0.24	3.70	0.52	-5.36	0.23	4.16	0.44	508	528	6.78	8.18

Table S3. Statistics of the anomalous diffraction data refinement for (^{tbs}L)Cr_3(NDipp) (**2**).

	Cr1				Cr2				Cr3				Reflections			
Energy (eV)	f'	esd	f''	esd	f'	esd	f''	esd	f'	esd	f''	esd	**Obs.**	**Total**	R_{obs}	wR_{all}
5967	-6.04	0.14	-0.31	0.92	-6.01	0.14	0.69	0.91	-6.01	0.15	2.07	0.76	653	899	5.11	5.34
5977	-6.58	0.14	2.00	0.70	-6.67	0.14	1.52	0.78	-6.67	0.14	0.17	0.88	652	906	4.80	5.03
5980	-6.65	0.14	0.77	1.40	-6.80	0.15	1.29	0.99	-6.80	0.14	0.78	1.30	659	905	5.14	5.38
5983	-6.92	0.14	1.20	0.93	-7.12	0.14	1.10	0.76	-7.12	0.14	1.86	0.80	665	904	4.92	5.08
5985	-7.21	0.14	1.60	0.80	-7.43	0.14	0.92	0.76	-7.43	0.14	1.35	0.86	681	905	5.20	5.63
5987	-7.50	0.14	2.21	0.62	-7.78	0.14	0.38	0.88	-7.78	0.14	0.51	0.91	659	905	5.08	5.31
5989	-7.87	0.14	1.01	0.86	-8.31	0.14	-0.20	0.85	-8.31	0.14	2.07	0.69	647	906	5.21	5.39
5991	-7.60	0.15	2.33	0.63	-8.36	0.15	1.57	0.62	-8.36	0.15	1.15	0.76	664	911	5.49	5.78
5993	-7.82	0.15	0.86	0.79	-8.55	0.15	1.55	0.57	-8.55	0.16	2.59	0.60	655	908	5.68	6.33
5995	-8.33	0.15	1.33	0.78	-8.58	0.15	1.89	0.55	-8.58	0.15	2.32	0.66	648	908	5.53	5.85
5997	-8.47	0.18	1.34	0.75	-8.63	0.19	3.22	0.50	-8.63	0.18	1.85	0.70	658	910	6.64	7.05
5999	-8.87	0.22	1.67	0.78	-8.55	0.23	3.39	0.53	-8.55	0.23	2.19	0.75	644	903	8.55	9.38
6001	-8.94	0.19	2.61	0.61	-8.36	0.20	3.47	0.48	-8.36	0.20	2.77	0.60	665	929	6.84	7.71
6003	-9.01	0.23	3.18	0.65	-8.59	0.24	3.65	0.56	-8.59	0.24	3.01	0.65	667	933	8.32	9.25
6005	-8.51	0.23	3.77	0.58	-8.27	0.24	4.45	0.50	-8.27	0.24	3.70	0.60	674	943	7.48	8.46
6007	-8.07	0.26	3.83	0.62	-7.86	0.27	5.10	0.52	-7.86	0.27	4.13	0.62	662	937	8.91	9.57
6009	-7.76	0.24	4.19	0.57	-6.76	0.25	5.07	0.50	-6.76	0.26	4.97	0.56	677	934	7.79	8.53
6011	-7.43	0.24	4.69	0.53	-6.58	0.24	4.85	0.50	-6.58	0.24	4.47	0.56	702	946	7.29	8.14
6014	-6.83	0.24	4.51	0.57	-6.24	0.24	4.57	0.53	-6.24	0.25	4.35	0.60	692	946	7.40	8.27
6017	-6.72	0.23	4.56	0.54	-6.03	0.23	4.53	0.50	-6.03	0.23	4.06	0.57	692	944	6.96	7.99
6027	-5.69	0.23	4.67	0.58	-5.61	0.24	4.20	0.57	-5.61	0.24	3.99	0.63	672	941	6.82	7.59

Table S4. Statistics of the anomalous diffraction data refinement for (^{tbs}L)Cr_3(μ^3–NBn) (**3**).

	**Cr1**				**Cr2**				**Cr3**				**Reflections**		R_{obs}	wR_{all}
Energy (eV)	f'	esd	f''	esd	f'	esd	f''	esd	f'	esd	f''	esd	**Obs.**	**Total**		
5967	-6.09	0.16	-0.05	0.42	-5.98	0.16	-0.05	0.52	-5.97	0.16	1.73	0.33	441	489	4.49	5.41
5977	-6.60	0.14	0.10	0.33	-6.61	0.15	0.07	0.39	-6.61	0.15	1.61	0.28	454	483	4.65	6.18
5981.5	-6.97	0.15	-0.24	0.41	-6.97	0.15	0.15	0.46	-7.00	0.16	1.70	0.33	446	482	4.59	5.59
5985	-7.34	0.15	-0.34	0.40	-7.43	0.15	-0.10	0.45	-7.49	0.16	1.58	0.33	450	483	4.75	5.74
5987	-7.68	0.15	1.66	0.39	-7.72	0.15	-0.06	0.46	-7.91	0.15	0.11	0.37	448	483	5.00	6.15
5989	-7.93	0.16	0.42	0.35	-8.51	0.16	0.84	0.42	-8.39	0.17	2.46	0.32	465	483	5.25	6.49
5991	-8.15	0.17	2.41	0.34	-7.68	0.17	0.26	0.39	-7.46	0.16	0.23	0.34	470	484	5.51	6.78
5993	-8.58	0.16	0.60	0.39	-8.22	0.17	2.16	0.35	-7.91	0.16	0.91	0.40	463	484	5.43	6.52
5995	-9.05	0.21	2.89	0.42	-8.71	0.21	0.62	0.49	-8.49	0.20	0.37	0.43	462	481	5.36	5.95
5997	-9.03	0.16	1.93	0.31	-8.53	0.17	2.26	0.36	-8.73	0.16	2.43	0.29	471	485	5.2	6.56
5999	-8.95	0.17	2.28	0.31	-8.37	0.18	2.84	0.35	-8.63	0.17	3.01	0.30	468	485	5.37	6.38
6001	-8.91	0.18	2.86	0.30	-8.29	0.19	2.93	0.32	-8.56	0.19	3.29	0.29	471	488	5.54	6.27
6003	-8.79	0.19	3.34	0.31	-8.33	0.20	3.49	0.33	-8.47	0.20	3.87	0.29	480	488	5.82	6.74
6005	-8.71	0.31	4.18	0.43	-7.91	0.32	3.88	0.45	-8.26	0.31	3.95	0.43	463	487	9.33	11.24
6008	-7.88	0.25	4.81	0.35	-7.68	0.25	4.29	0.36	-7.97	0.25	4.88	0.33	467	489	6.84	8.12
6011	-6.96	0.27	4.83	0.40	-7.22	0.27	4.46	0.41	-7.15	0.27	5.13	0.37	458	487	7.01	7.95
6017	-6.08	0.24	4.31	0.32	-6.32	0.25	4.55	0.35	-6.28	0.25	5.28	0.30	477	488	6.36	7.90
6027	-5.52	0.26	3.98	0.39	-5.31	0.26	4.08	0.39	-5.10	0.26	4.44	0.36	475	494	6.87	8.19

Table S5. Statistics of the anomalous diffraction data refinement for ([tbs]L)Cr_3(μ^3–NPh)(NPh) (**5**).

	**Cr1**				**Cr2**				**Cr3**				**Reflections**		R_{obs}	wR_{all}
	f'	esd	f''	esd	f'	esd	f''	esd	f'	esd	f''	esd	**Obs.**	**Total**		
5967	-6.01	0.20	0.32	0.79	-6.23	0.20	1.94	0.69	-6.01	0.19	0.11	0.73	392	423	5.90	7.66
5977	-6.57	0.15	0.62	0.70	-6.59	0.16	1.15	0.78	-6.63	0.15	0.68	0.81	499	551	5.44	7.07
5980	-6.82	0.18	1.02	0.69	-7.08	0.18	1.68	0.65	-6.91	0.17	0.41	0.65	530	584	6.47	8.37
5983	-6.93	0.15	0.56	0.48	-7.22	0.15	1.49	0.44	-7.11	0.14	0.21	0.47	539	585	6.16	8.40
5985	-7.06	0.15	0.65	0.43	-7.43	0.16	1.54	0.43	-7.32	0.15	0.04	0.44	541	586	6.28	9.12
5987	-7.28	0.15	0.83	0.47	-7.84	0.16	1.50	0.47	-7.76	0.15	0.09	0.49	537	582	6.57	8.93
5989	-7.58	0.16	0.85	0.45	-8.44	0.16	2.02	0.43	-8.34	0.16	0.63	0.48	540	584	6.21	8.54
5991	-7.73	0.16	1.06	0.38	-7.92	0.16	1.98	0.37	-7.56	0.15	0.91	0.41	546	582	6.53	9.14
5993	-8.03	0.15	1.18	0.40	-8.31	0.15	1.71	0.40	-7.84	0.15	0.60	0.41	546	582	6.3	9.06
5995	-8.46	0.15	1.21	0.35	-8.83	0.16	2.49	0.36	-8.50	0.15	1.22	0.38	551	585	6.36	8.68
5997	-9.03	0.16	1.57	0.34	-8.58	0.17	2.86	0.36	-8.60	0.16	1.59	0.36	554	586	6.64	8.56
5999	-9.49	0.18	2.24	0.42	-8.50	0.19	3.14	0.44	-8.73	0.18	2.04	0.43	545	585	7.05	8.54
6001	-9.38	0.19	2.81	0.40	-8.37	0.20	3.34	0.42	-8.73	0.19	2.61	0.41	547	586	7.34	8.79
6003	-9.25	0.20	3.70	0.34	-8.25	0.20	3.72	0.35	-8.52	0.19	3.17	0.34	556	584	7.34	9.09
6005	-8.74	0.25	4.40	0.44	-8.24	0.25	3.93	0.44	-8.58	0.24	3.55	0.43	557	590	8.44	10.18
6007	-7.89	0.23	4.53	0.35	-7.85	0.23	4.21	0.36	-8.43	0.22	4.17	0.35	561	590	7.85	9.66
6009	-7.35	0.25	4.66	0.37	-7.53	0.25	4.67	0.39	-7.81	0.25	4.78	0.38	567	588	8.33	10.15
6011	-6.99	0.26	4.61	0.39	-7.20	0.26	4.64	0.42	-7.25	0.25	4.78	0.40	555	586	8.29	10.10
6014	-6.91	0.22	4.32	0.30	-6.69	0.23	4.84	0.32	-6.79	0.22	4.71	0.30	567	586	7.60	9.61
6017	-6.79	0.25	4.52	0.37	-6.40	0.25	4.85	0.39	-6.27	0.25	4.72	0.37	562	589	8.14	10.09
6027	-5.77	0.25	4.39	0.43	-5.51	0.26	4.35	0.46	-5.54	0.25	4.21	0.43	546	585	7.62	9.34

Table S6. Comparison of relevant structural metrics. All bond metrics are reported in Å.

(^{tbs}L)Cr_3(CNBn) (**4**)		(^{tbs}L)Cr_3(NDipp) (**2**)		(^{tbs}L)Cr_3(μ^3–NBn) (**3**)		(^{tbs}L)Cr_3(μ^3–NPh)(NPh) (**5**)	
Cr1-Cr2	2.5541(6)	Cr1-Cr2	2.7034(7)	Cr1-Cr2	2.7547(7)	Cr1-Cr2	2.9768(7)
Cr2-Cr3	2.6715(5)	Cr2-Cr3	2.5567(6)	Cr2-Cr3	2.5977(6)	Cr2-Cr3	2.6867(6)
Cr3-Cr1	3.0128(6)	Cr3-Cr1	2.8937(6)	Cr3-Cr1	2.6847(8)	Cr3-Cr1	2.8247(7)
Cr1-C43	2.031(2)	Cr1-N7	1.681(2)	Cr1-N7	1.927(3)	Cr1-N8	2.030(3)
Cr1-N1	1.9188(18)	Cr1-N1	1.9828(19)	Cr2-N7	1.954(2)	Cr2-N8	1.9467(19)
Cr1-N2	2.0817(18)	Cr1-N2	2.0504(19)	Cr3-N7	2.005(2)	Cr3-N8	1.923(2)
Cr1-N5	2.1802(19)	Cr1-N5	2.0561(19)	Cr1-N1	1.961(2)	Cr1-N7	1.691(3)
Cr2-N3	2.0924(19)	Cr2-N3	1.9930(19)	Cr1-N2	1.943(2)	Cr1-N5	2.064(2)
Cr2-N4	2.0298(19)	Cr2-N4	2.002(2)	Cr1-N3	2.057(2)	Cr1-N6	2.049(2)
Cr2-N1	2.1202(19)	Cr2-N1	2.0987(19)	Cr2-N3	1.955(3)	Cr1-N1	2.251(2)
Cr2-N2	2.3309(19)	Cr2-N2	2.199(2)	Cr2-N4	1.990(2)	Cr2-N1	1.977(3)
Cr3-N5	2.0378(19)	Cr3-N5	2.0137(19)	Cr2-N5	2.034(2)	Cr2-N2	1.907(2)
Cr3-N6	1.8909(17)	Cr3-N6	1.9788(19)	Cr2-C19	2.488(3)	Cr2-N3	2.035(2)
Cr3-N3	1.9467(16)	Cr3-N3	2.0318(19)	Cr3-N5	2.024(2)	Cr3-N3	1.9544(19)
Cr3-C13	2.374(2)	Cr3-C13	2.410(3)	Cr3-N6	1.993(3)	Cr3-N4	1.961(3)
				Cr3-N1	2.049(3)	Cr3-N5	2.012(3)
						Cr3-C19	2.428(3)

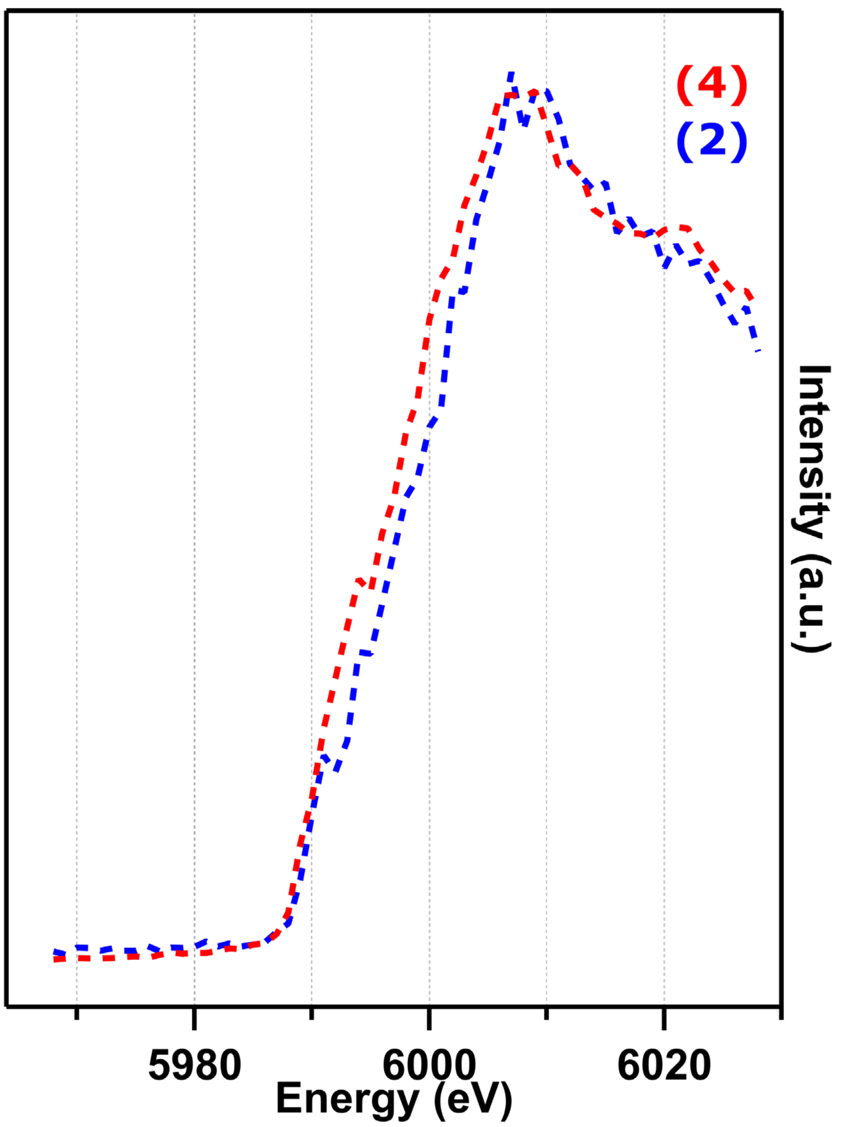

Figure S1. Fluorescence scans for **4** (red) and **2** (blue).

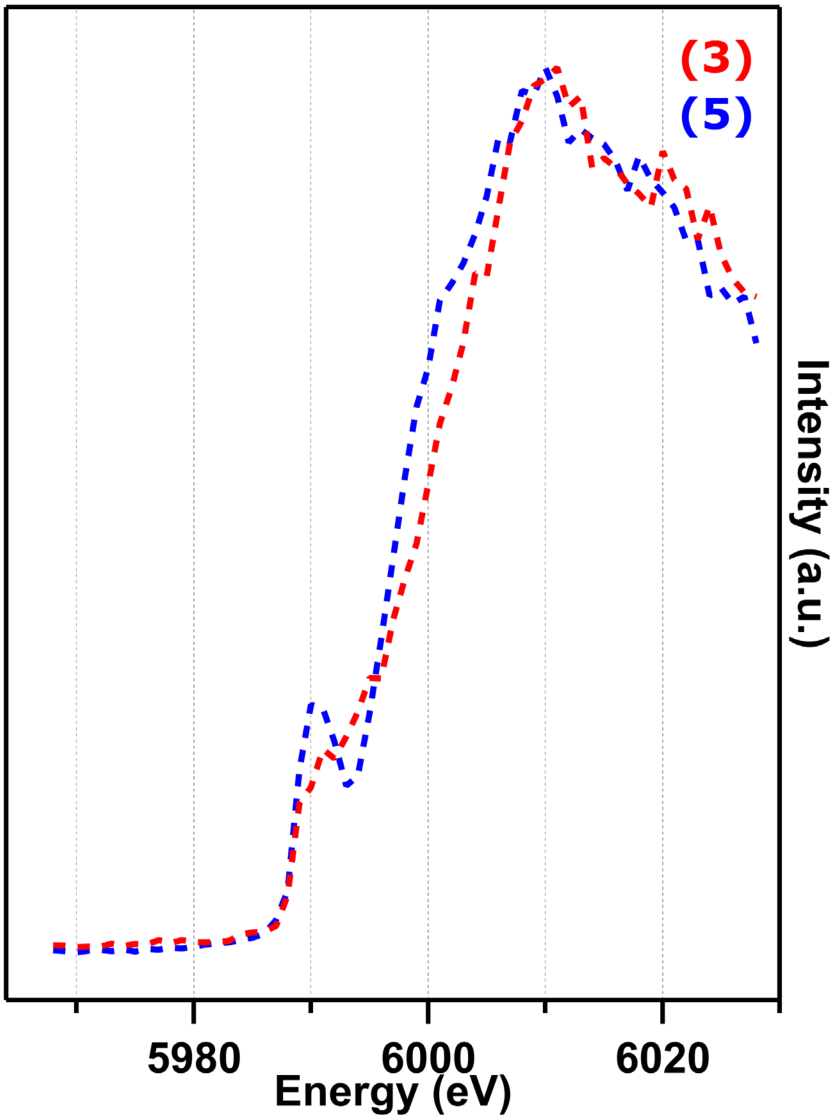

Figure S2. Fluorescence scans for **3** (red) and **5** (blue).

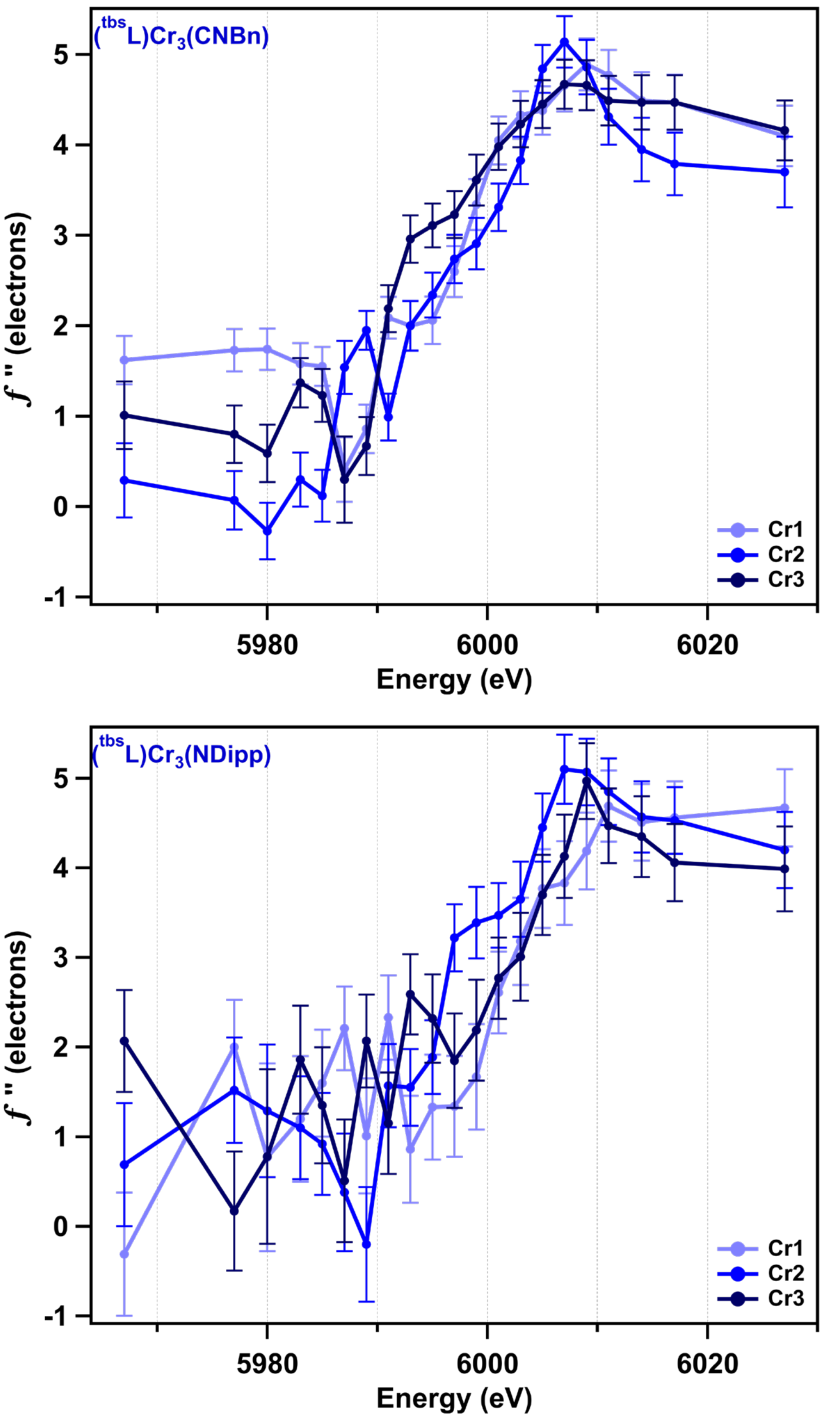

Figure S3. Anomalous scattering f'' plots for **4** (top) and **2** (bottom).

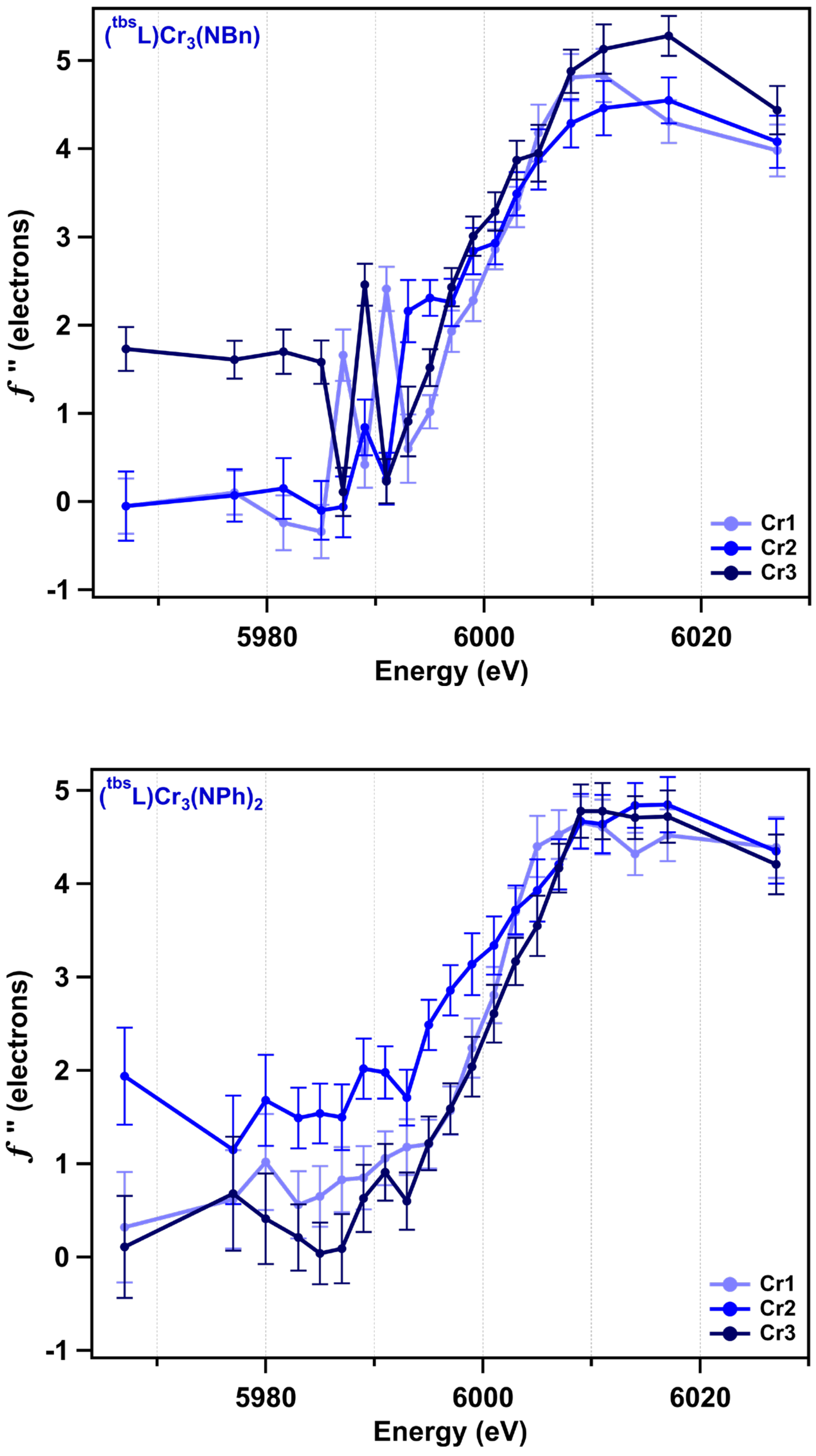

Figure S4. Anomalous scattering f'' plots for **3** (top) and **5** (bottom).

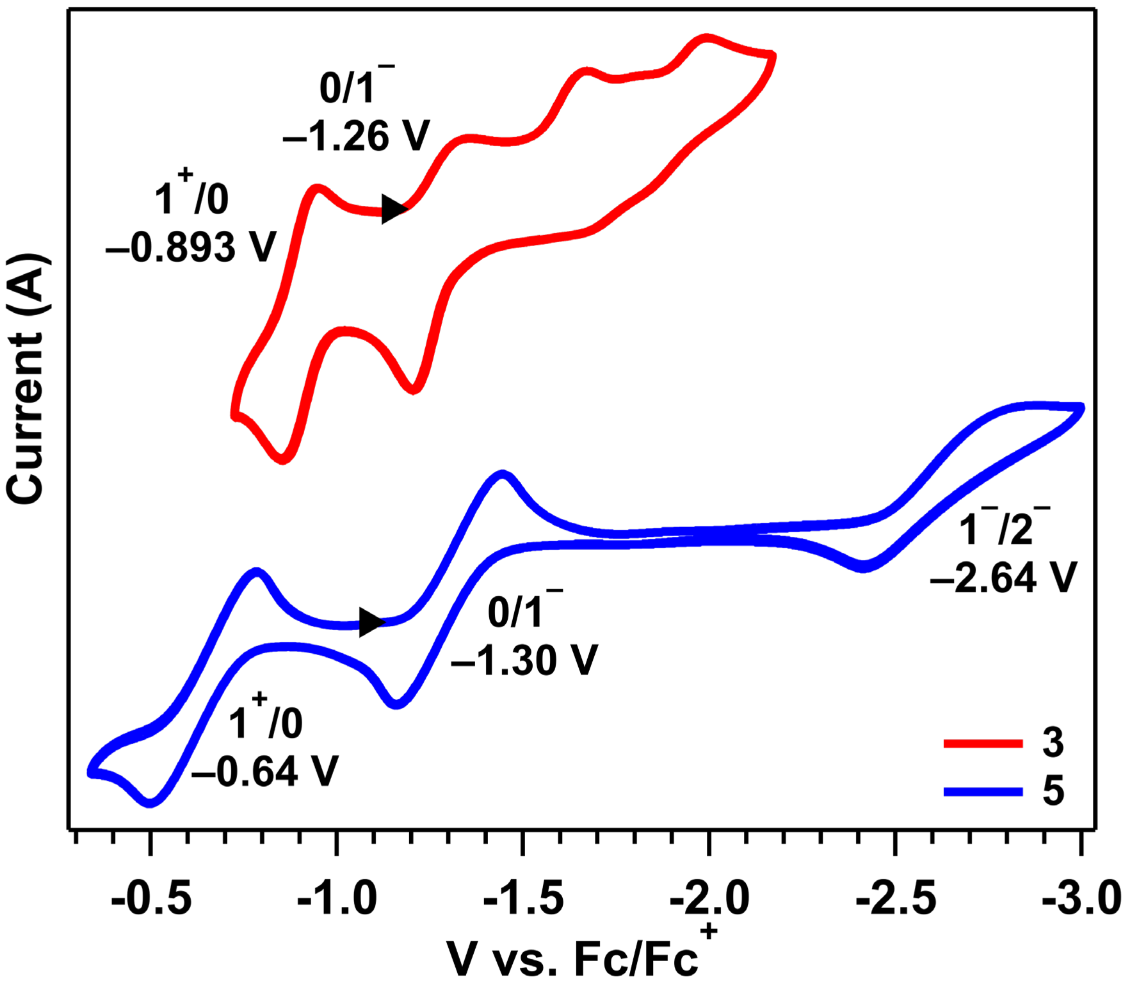

Figure S5. Cyclic voltammograms of **3** and **5** in 0.1 M $[NBu_4][PF_6]$ in THF at a 0.05 V/s scan rate, referenced to ferrocene$^{0/+}$.

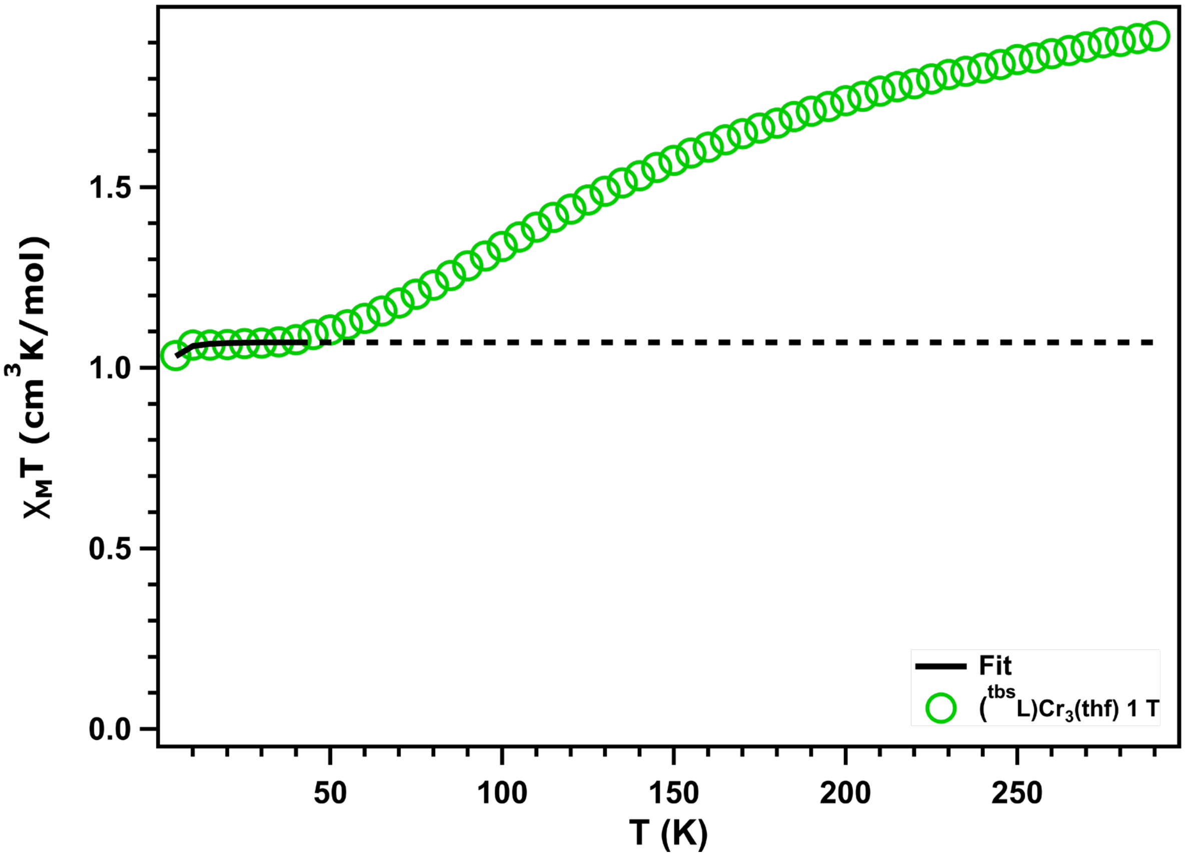

Figure S6. Variable-temperature magnetic susceptibility of **1**. Data collected on heating from 5 to 292 K. A fit of the ground spin state is shown in solid black, while a linear extrapolation of this fit to higher temperatures in shown as a dashed black line. The fit corresponds to a single spin system with S=1, g = 2.07, D = –3.14, and E = –0.01.

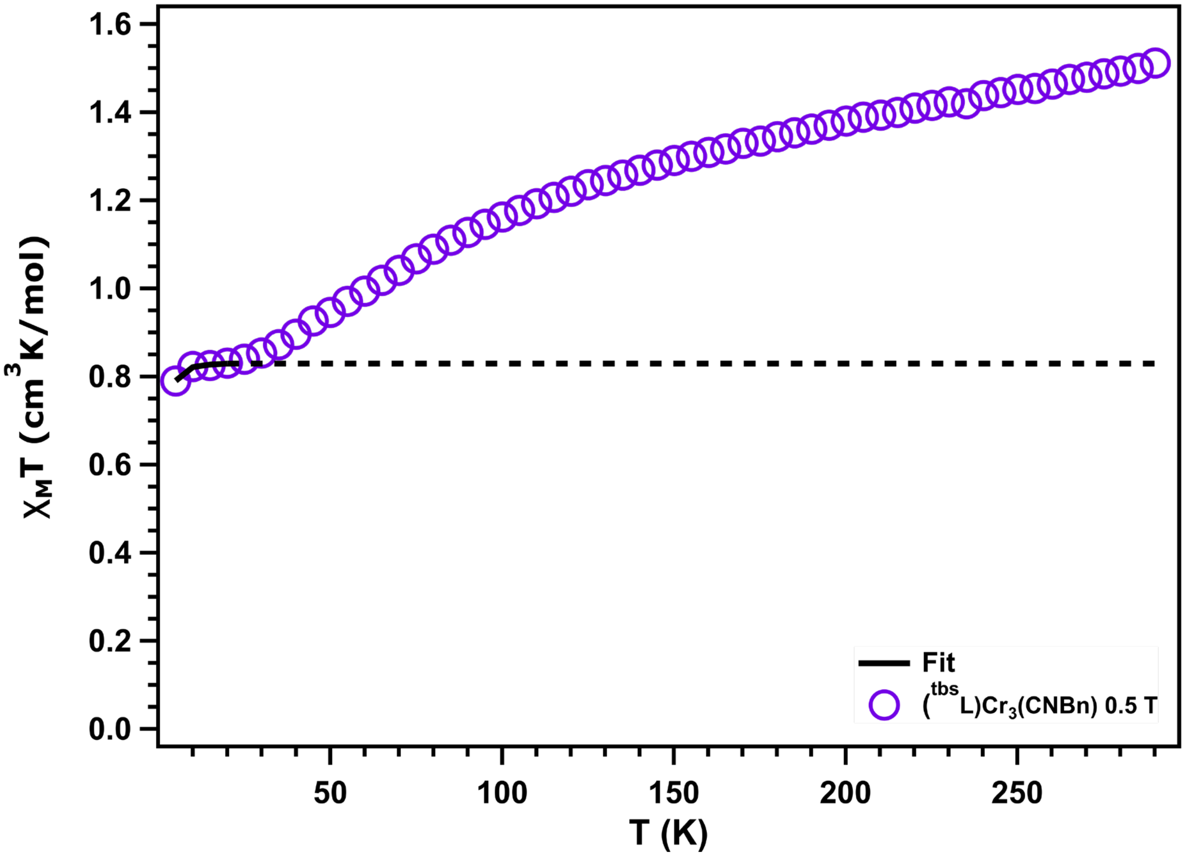

Figure S7. Variable-temperature magnetic susceptibility of **4**. Data collected on heating from 5 to 292 K. A fit of the ground spin state is shown in solid black, while a linear extrapolation of this fit to higher temperatures in shown as a dashed black line. The fit corresponds to a single spin system with S=1, g=1.82, and D=3.17.

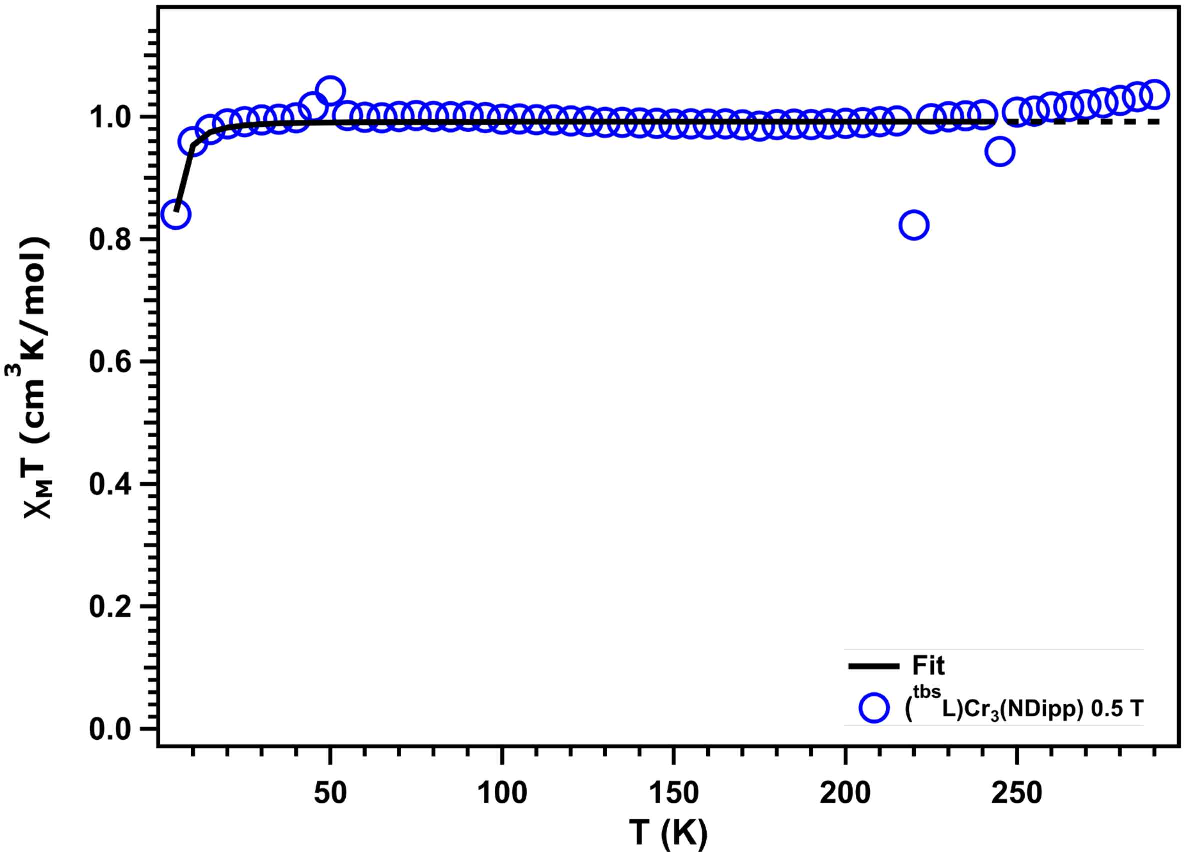

Figure S8. Variable-temperature magnetic susceptibility of **2**. Data collected on heating from 5 to 292 K. A fit of the ground spin state is shown in solid black, while a linear extrapolation of this fit to higher temperatures in shown as a dashed black line. The fit corresponds to a single spin system with S=1, g = 1.99, and D = 5.64. The peak near 50 K is caused by a minor impurity of oxygen in the sample chamber. These points were excluded from the fit, as were the two data points at higher temperature which have anomalously low values due to instrument error.

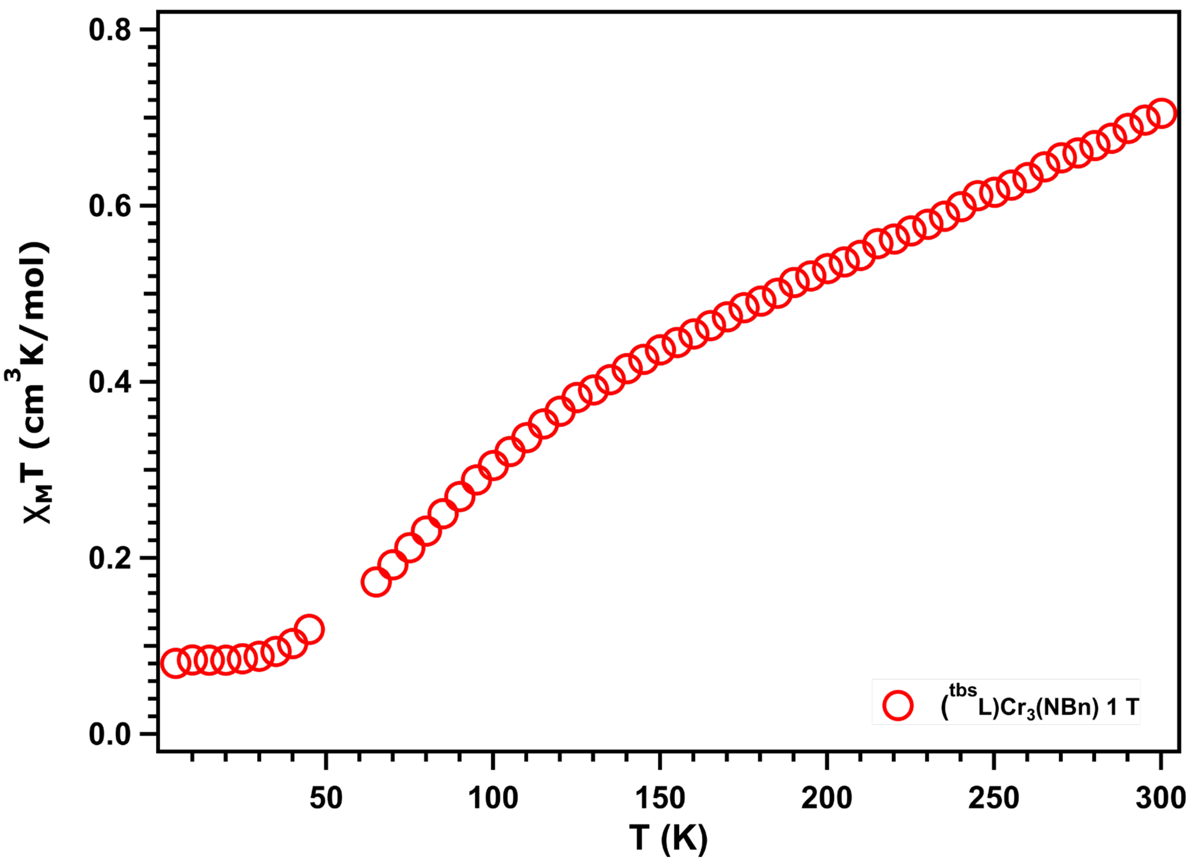

Figure S9. Variable-temperature magnetic susceptibility of **3**. Data collected on heating from 5 to 300 K. Note that the expected spin-only value of the magnetic susceptibility for a triplet ground state is 1 while the expected value for a singlet ground state is 0. The missing points have highly negative values (-5 to -7) due to instrument error.

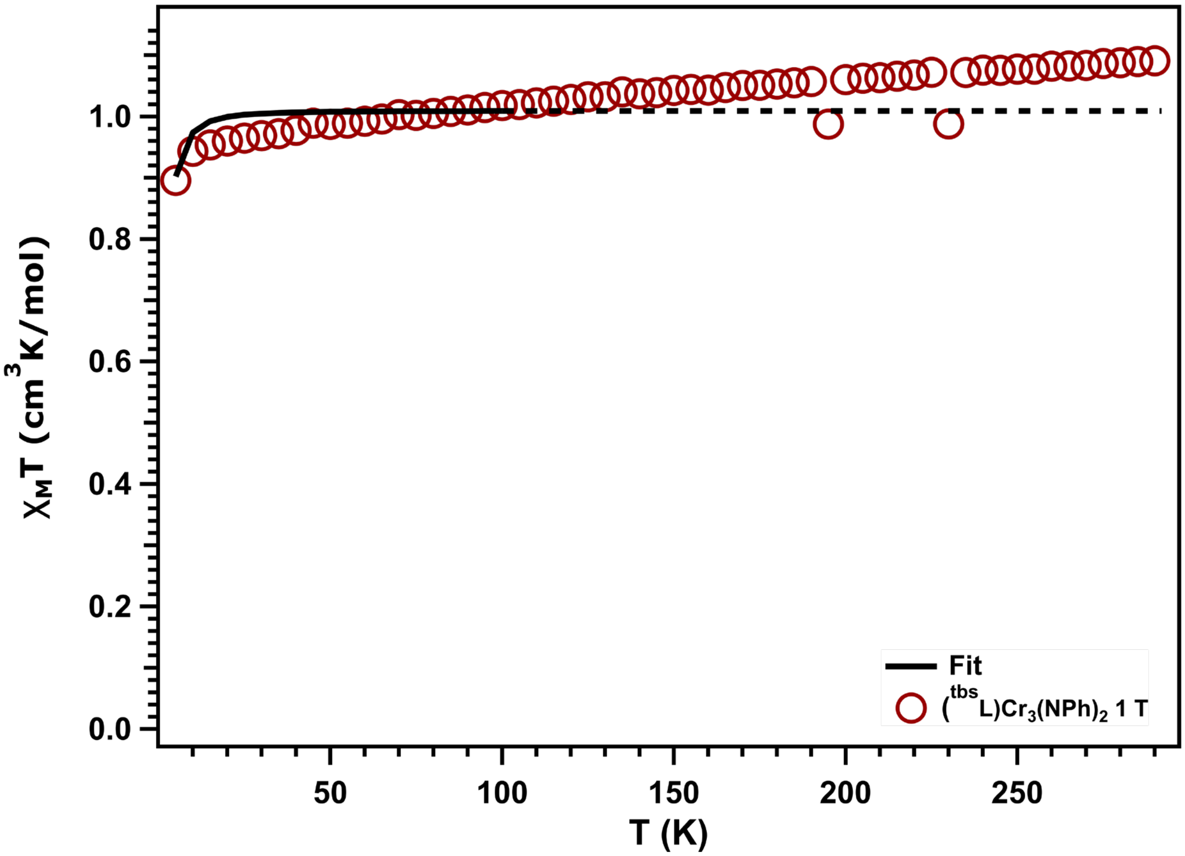

Figure S10. Variable-temperature magnetic susceptibility of **5**. Data collected on heating from 5 to 292 K. A fit of the ground spin state is shown in solid black, while a linear extrapolation of this fit to higher temperatures in shown as a dashed black line. The fit corresponds to a single spin system with S=1, g = 2.01, and D = –6.10.

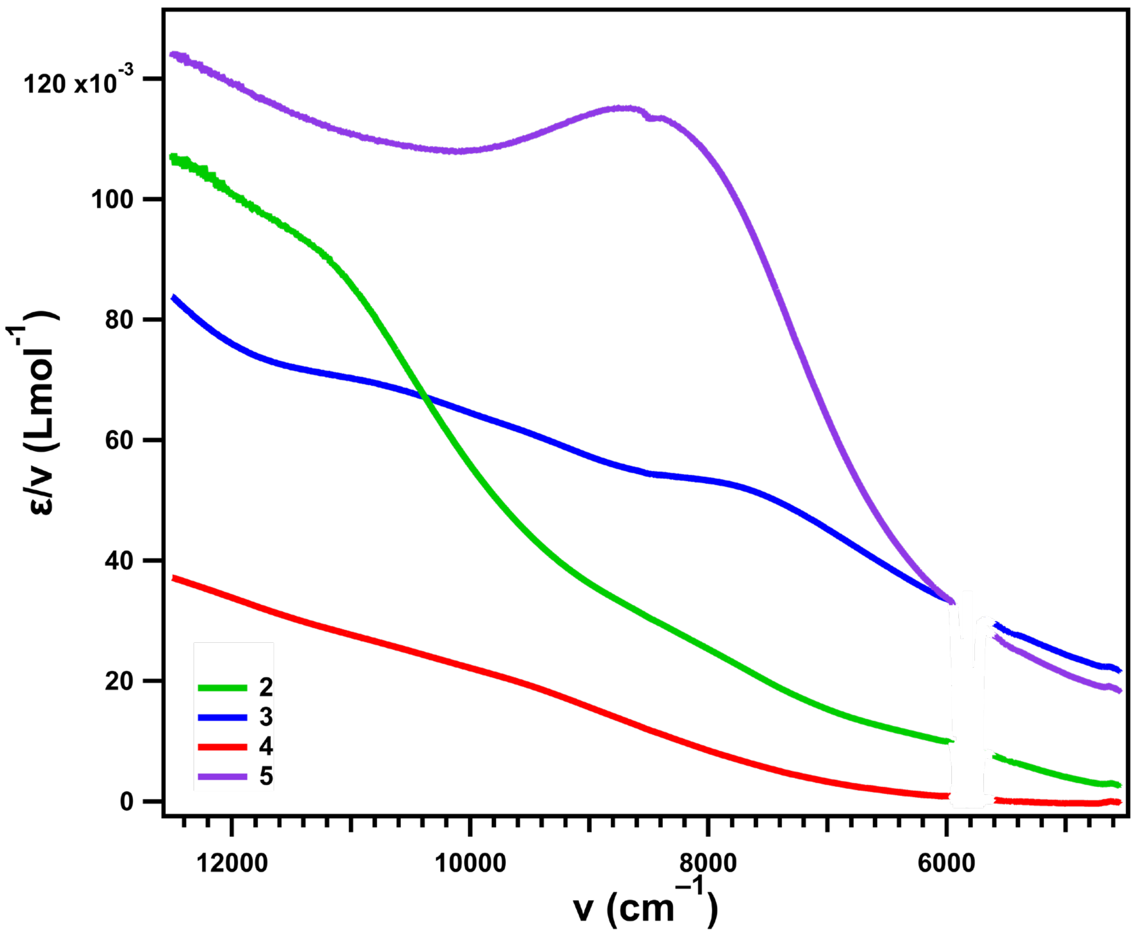

Figure S11. UV-Vis/Near-IR spectra of **2** (green), **3** (blue), **4** (red), and **5** (purple) at 298 K in THF. The region between 5800 and 6000 cm^{-1} contains a strong signal from THF solvent background that saturated the detector and has therefore been omitted.

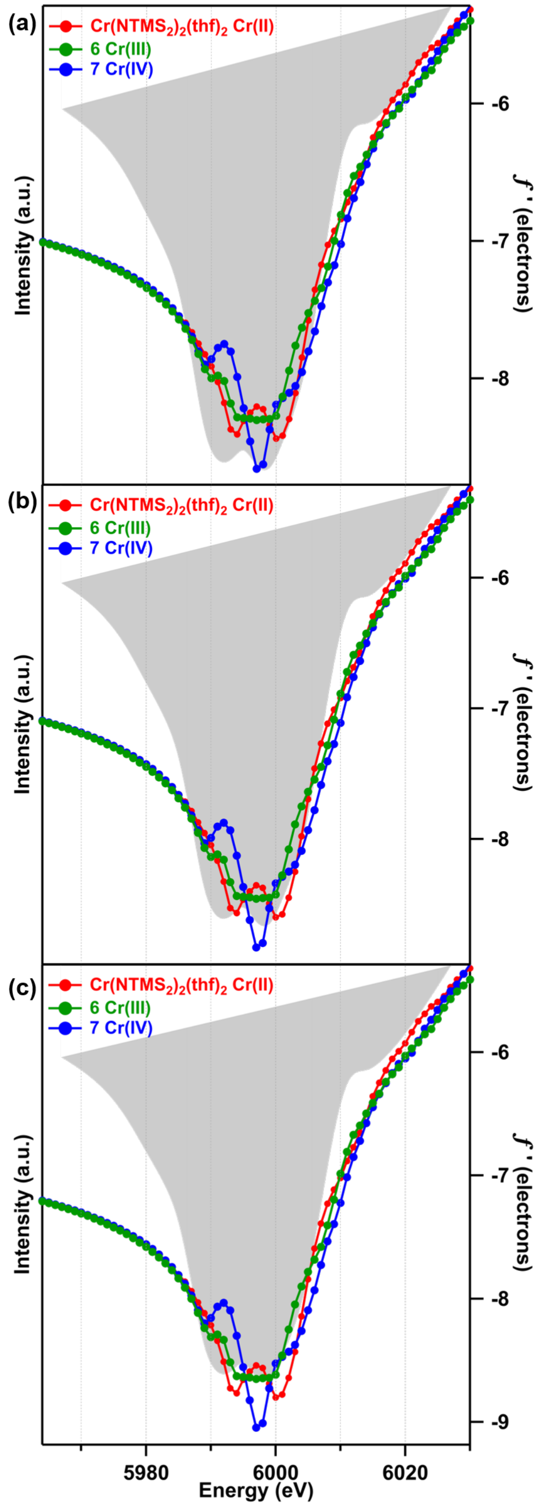

Figure S12. Overalay of edge-scan derived predicted *f'* spectra for mononuclear references with the average *f'* of (^{tbs}L)Cr_3(CNBn) (gray). Intensity of the gray trace was set to match the minimum of the trace for the Cr(IV), Cr(III), and Cr(II) references, respectively, in (a)-(c). This is included to demonstrate that due to the arbitrary intensity of the predicted *f'* spectra it is not possible to productively compare the location of the falling or rising edges between these predicted *f'* spectra and the experimentally collected *f'* data.

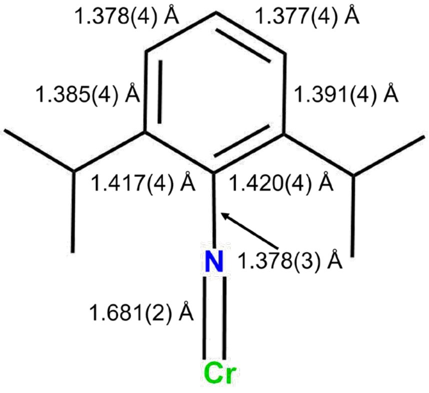

Figure S13. Bond metrics for the imido fragment of **2**.

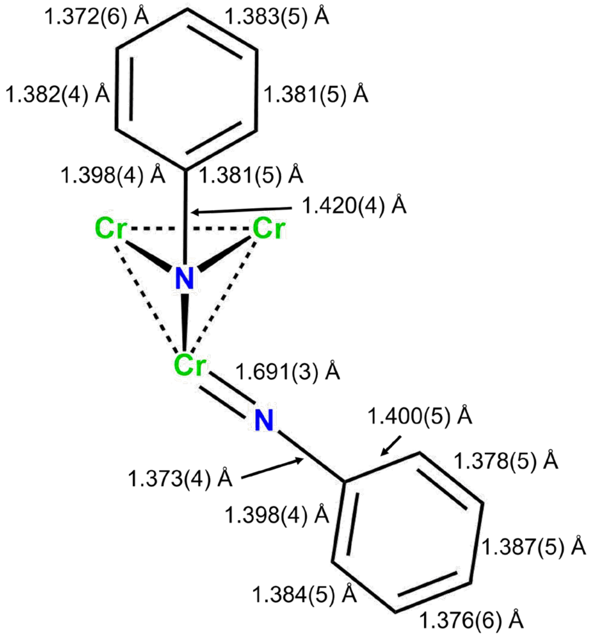

Figure S14. Bond metrics for the imido fragments of **5**.

References Cited

[1] N. F. Chilton, R. P. Anderson, L. D. Turner, A. Soncini, K. S. Murray, *J. Comput. Chem.* **2013**, *34*, 1164-1175.

[2] Scheldrick, G. M. SADABS, Version 2.03; Bruker Analytical X-Ray Systems, Inc.: Madison, WI, 2000.

[3] G. M. Sheldrick, *Acta Cryst. C* **2015**, *71*, 3-8.

[4] O. V. Dolomanov, L. J. Bourhis, R. J. Gildea, J. A. K. Howard, H. Puschmann, *J. Appl. Crystallogr.* **2009**, *42*, 339-341.

[5] Spek, A. L. PLATON, A Multipurpose Crystallographic Tool; Utrecht University: Utrecht, The Netherlands, 2010.
